# Supplementary material for: Mathematical modelling of the impact of expanding levels of malaria control interventions on Plasmodium vivax
Source: Nat Commun. 2018 Aug 17;9:3300. doi: 10.1038/s41467-018-05860-8 (PMC6097992; doi:10.1038/s41467-018-05860-8)
Supplement: Supplementary file 1 — Supplementary Information [file 41467_2018_5860_MOESM1_ESM.pdf]

Mathematical modelling of the impact of expanding levels of malaria control interventions on *Plasmodium vivax*

Michael White, Patrick Walker, Stephan Karl, Manuel W. Hetzel, Tim Freeman,
Andreea Waltmann, Moses Laman, Leanne Robinson, Azra Ghani, Ivo Mueller

Supplementary Information file: Mathematical details of the transmission model, assumed and estimated parameter values, results of inter-provincial transmission model, and supplementary figures with additional model simulations.

1. Supplementary methods: Mathematical description of *Plasmodium vivax* transmission model

There is a rich tradition of studying the transmission dynamics of *Plasmodium falciparum* parasites between humans and mosquitoes using Ross-Macdonald models[1,2]. These models split the human and mosquito populations into compartments according to whether they are susceptible or infected, with the transition rates between these compartments being described by a system of differential equations. These models have been reviewed extensively in the literature[3,4]. In order to analyse the transmission dynamics of *P. vivax* in the Ross-Macdonald framework, we need to include a representation of relapse infections[5]. Here we describe how a Ross-MacDonald style model of *P. vivax* transmission[5,6] can be extended to give a model that can be calibrated against a range of epidemiological data sets. This is done by including a more detailed representation of blood-stage *P. vivax* (section 1.1); accounting for human age structure (1.2) and heterogeneity in exposure to mosquito bites (1.3); relapses (1.4); the acquisition of immunity (1.5); and a model of mosqutio population dynamics accounting for larval stages (1.7). Notably, the model is described below in its deterministic format which is utilised for fitting to data. An equivalent, stochastic individual-based formulation of the model is also developed to allow simulation of combinations of interventions.

1.1. Model of blood-stage *P. vivax* dynamics in humans

A large proportion of blood-stage *P. vivax* infections have very low parasite density, often remaining asymptomatic and undetectable via light microscopy. In order to provide a parsimonious representation of *P. vivax* blood-stage infection that accords with routinely collected epidemiological data, we represent blood-stage *P. vivax* via three states:

- I_{PCR} : sub-microscopic blood-stage infection not detectable by routine light microscopy, but detectable by laboratory-based PCR methods.
- I_{LM} : patent blood-stage infection detectable by light microscopy
- I_{D} : clinical disease – *P. vivax* parasitaemia accompanied by fever

The exact definition of the above categories depends on the epidemiological and laboratory methods used for detecting parasites and measuring parasite density. For example, if two studies measure *P. vivax* prevalence by light microscopy (*Pv*PR_{LM}), the measurements may not be directly comparable if substantially different microscopy protocols were used. Similarly, the definition of a clinical episode of *P. vivax* is important. The definition of a clinical episode utilised in our model is constructed to match the data routinely collected in epidemiological studies where a clinical episode is defined as fever ≥ 38 °C in the last 48 hours with *P. vivax* parasite density ≥ 500/mL. All of the calibration data on *P. vivax* prevalence and incidence were collected using the same methodology and were overseen by the same teams based in the Papua New Guinea Institute of Medical Research (PNGIMR) and the Walter and Eliza Hall Institute (WEHI).

In addition to the infection states described above, we include two states that account for the effects of anti-malarial treatment. In particular, we assume that a proportion χ_T of new episodes of clinical malaria receive prompt treatment with a blood-stage anti-malarial following the onset of symptoms. During this period *T*, individuals are still infectious but parasites are being rapidly cleared. Following clearance of parasitaemia, individuals enter a period of prophylaxis, *P*, where they are protected from new blood-stage infections, before finally returning to the fully susceptible state *S*. The model is described by the system of partial differential equations below as well as the schematic in Supplementary Figure 1.

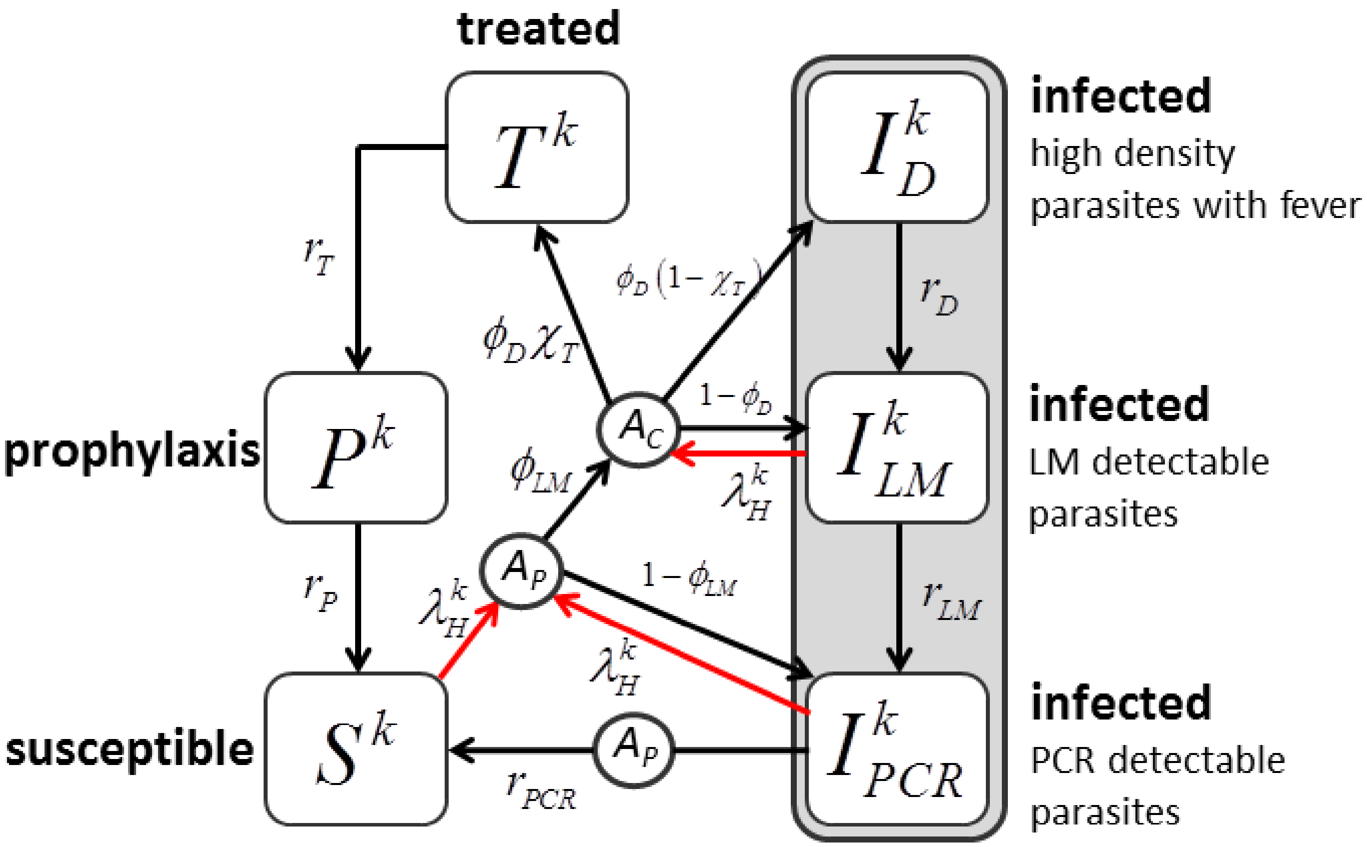

Supplementary Figure 1: Compartmental representation of *P. vivax* transmission model in humans. Infected individuals can be in one of three compartments depending on whether blood-stage parasitaemia is detectable by PCR (I_{PCR}), light microscopy (I_{LM}) or has high density with accompanying fever (I_D). A proportion of individuals that progress to a symptomatic episode of *P. vivax* will be treated by a blood-stage drug (*T*) leading to clearance of blood-stage parasitaemia and a period of prophylactic protection (*P*) before returning to the susceptible state (*S*). The superscript *k* denotes the number of batches of relapse causing hypnozoites in the liver. Transitions between compartments occur at the rates shown, defined in supplementary equation (1) below. Each square denotes a compartment and the circles denote the dependence of transition rates between compartments on levels of anti-parasite immunity (A_P) and levels of clinical immunity (A_C). Red arrows denote new blood-stage infections arising from either new mosquito bites or relapses.

The system of differential equations below describes the transitions between the compartments in Supplementary Figure 1. Note that at this stage we do not yet account for relapses and the force of infections on humans is entirely due to new mosquito bites $\lambda_H^0(t-d_E)$, where d_E is the latent period of 10 days to account for the time between mosquito inoculation of sporozoites, development of sporozoites into merozoites within the liver and the first detection of blood-stage merozoites. This model is therefore directly comparable to the compartmental version of the *P. falciparum* in Griffin *et al.*[7], which is used as the starting point for *P. vivax* model development.

$$\begin{aligned}
\frac{dS}{dt} &= -\lambda_H^0(t-d_E)S + r_{PCR}I_{PCR} + r_P P \\
\frac{dI_{PCR}}{dt} &= -\lambda_H^0(t-d_E)I_{PCR} + \lambda_H^0(t-d_E)(1-\phi_{LM})(S+I_{PCR}) - r_{PCR}I_{PCR} + r_{LM}I_{LM} \\
\frac{dI_{LM}}{dt} &= -\lambda_H^0(t-d_E)I_{LM} + \lambda_H^0(t-d_E)\phi_{LM}(1-\phi_D)(S+I_{PCR}) + \lambda_H^0(t-d_E)(1-\phi_D)I_{LM} - r_{LM}I_{LM} + r_D I_D \\
\frac{dI_D}{dt} &= +\lambda_H^0(t-d_E)\phi_{LM}\phi_D(1-\chi_T)(S+I_{PCR}) + \lambda_H^0(t-d_E)\phi_D(1-\chi_T)I_{LM} - r_D I_D \\
\frac{dT}{dt} &= +\lambda_H^0(t-d_E)\phi_{LM}\phi_D\chi_T(S+I_{PCR}) + \lambda_H^0(t-d_E)\phi_D\chi_T I_{LM} - r_T T \\
\frac{dP}{dt} &= +r_T T - r_P P
\end{aligned} \tag{1}$$

Supplementary equation (1) implies that in the absence of new infections, the time spent by an individual in a compartment is exponentially distributed with mean duration $1/r_{PCR}$ (I_{PCR}), $1/r_{LM}$ (I_{LM}), $1/r_D$ (I_D), $1/r_T$ (T) and $1/r_P$ (P). Furthermore, there are three factors which determine whether a new blood-stage infection will have low density and only be detectable by PCR (I_{PCR}), become detectable by light microscopy (I_{LM}), result in an untreated clinical episode of *P. vivax* malaria (I_D), or a treated clinical episode of *P. vivax* malaria (T):

- ϕ_{LM}: the probability that a blood-stage infection will become detectable by light microscopy
- ϕ_D: the probability that a blood-stage infection that is detectable by light microscopy will progress to cause an episode of clinical malaria
- χ_T: the proportion of symptomatic episodes of clinical *P. vivax* that receive blood-stage treatment

The model represented in supplementary equation (1) does not account for ageing and human demography, heterogeneity in exposure to mosquito bites, the acquisition of immunity, or relapses. The first two of these factors are included in the model in the same as for the *P. falciparum* model in Griffin *et al.*[7]. For completeness these are included here in sections 1.2 and 1.3. The third factor builds on the *P. falciparum* model but is extended to incorporate relapses in section 1.5. The fourth factor (relapse) is specific to *P. vivax* and hence newly developed in section 1.4.

1.2. Human demography and age-dependent exposure to mosquito bites

Human demography

We assume that all humans are subject to a constant death rate μ_H. This assumption is consistent with demographic data from developing countries with high birth and death rates. We assume that the population birth rate is μ_H so that the total population size is balanced over time. Finally we assume a maximum age a_{max}. The distribution of ages in the population can therefore be described by the following truncated exponential distribution:

$$W(a) = \frac{\mu_H e^{-\mu_H a}}{1-e^{-\mu_H a_{\max}}} \tag{2}$$

Age-dependent exposure to mosquito bites

It has been repeatedly observed that an individual's exposure to mosquito bites will vary over the course of their lifetime, with adults receiving substantially more bites than children[8,9]. This is due to several reasons including the larger surface area of adults and the tendency of adults to spend a greater proportion of time outdoors during the evening. In particular, we assume that age-dependent exposure to mosquito bites takes the following functional form:

$$X(a) = \frac{1}{\omega_{\text{age}}}\left(1 - \rho_{age} e^{-\frac{a}{a_0}}\right) \quad (3)$$

where ω_{age} is a normalising constant calculated to ensure:

$$\int_0^{a_{\max}} X(a)W(a) = 1$$
$$\Rightarrow \omega_{\text{age}} = 1 - \frac{\rho_{\text{age}}\mu_H a_0}{\mu_H a_0 + 1} \frac{1 - e^{-\left(\mu_H + \frac{1}{a_0}\right)a_{\max}}}{1 - e^{-\mu_H a_{\max}}} \quad (4)$$

1.3. Heterogeneity in exposure to mosquito bites

Heterogeneity in exposure to mosquito bites occurs across all spatial scales with differences in biting rates observed between villages, between houses within villages, and between individuals in houses[10]. This heterogeneity may arise due to variation in several factors including, but not limited to, attractiveness of humans to mosquitoes, housing standards, socio-economic status, and distance from mosquito breeding sites. We assumed that the heterogeneity in exposure can be described by a log-Normal distribution[11]. In particular, if the entomological inoculation rate (EIR) in a community is ε, then we assume that an individual's exposure is $\zeta\varepsilon$ where ζ follows a log-Normal distribution defined as follows:

$$W(\zeta) = \frac{1}{\zeta\sigma\sqrt{2\pi}} e^{-\frac{\log(\zeta)^2}{2\sigma^2}} \quad (5)$$

The mean exposure is thus ε with standard deviation $\varepsilon\sqrt{e^{\sigma^2} - 1}$.

In a region with EIR = ε, the age and exposure dependent force of infection from mosquito bites will therefore be

$$\lambda_H^0(a,\zeta) = \frac{1}{\omega_{\text{age}}}\left(1 - \rho_{age} e^{-\frac{a}{a_0}}\right)\zeta\varepsilon \quad (6)$$

In this equation for the force of infection, the superscript 0 denotes that relapses are not accounted for. The age and exposure dependent distribution of individuals is then given by:

$$W(a,\zeta) = W(a)W(\zeta) \quad (7)$$

Note that although an individual's age will change over time, we assume that their level of heterogeneity in exposure to mosquito bites is constant over time.

1.4. Incorporation of relapses

A bite from a *P. vivax* infectious mosquito may cause an initial episode of blood-stage infection, and may also lead to the formation of liver-stage hypnozoites which lie dormant in the liver for weeks to years before relapsing to cause new blood-stage infections. The dynamics of hypnozoite infection and relapses can be accounted for using models with a range of complexity, with more complex models facilitating a greater degree of realism. Here we provide an overview of three approaches of increasing complexity[5,6]:

1. **Binary hypnozoite model.** Individuals can be in one of two states: infected or not infected with hypnozoites. Infected individuals experience relapses at rate f and clear hypnozoites at rate γ_L. There is no role for super-infection, i.e. individuals cannot be infected more than once.
2. **Batch hypnozoite model.** Similar to the above model except that the process of super-infection allows multiple batches of hypnozoites from different mosquito bites to be accumulated. Thus an individual may have k batches of hypnozoites. Hypnozoites from any batch may activate giving a relapse rate of kf. Each batch is subject to clearance at a constant rate so that the number of batches reduces from k to k - 1 at rate $k\gamma_L$.
3. **Hypnozoite density model.** The number of hypnozoites in an individual's liver is explicitly modelled. A new mosquito bite introduces a batch with a variable number of hypnozoites. Each hypnozoite can either activate to cause a relapse or die. Liver clearance occurs when all individual hypnozoites have either activated or died.

For the batch hypnozoite model, it is necessary to limit the maximum number of batches to some value K. In particular, we find that the binary hypnozoite model is nested within the batch hypnozoite model by setting K = 1. The hypnozoite density model requires the number of hypnozoites to be explicitly accounted for[6], incurring substantial computational cost, thus limiting its use in more detailed models of *P. vivax* transmission dynamics. It is thus not considered further. In the following, we provide the mathematical details of the batch hypnozoite model, noting that the binary hypnozoite model can be regained by setting K = 1.

We assume that $Z_H^k(t,a,\zeta)$ is the proportion of a population at time t, of age a, with exposure ζ that has k batches of hypnozoites. If the maximum number of batches of hypnozoites is K, then the dynamics of the hypnozoite reservoir can be described by the following system of partial differential equations:

$$\begin{aligned}
\frac{\partial Z_H^0}{\partial t}+\frac{\partial Z_H^0}{\partial a}+\frac{\partial Z_H^0}{\partial \zeta} &= -\lambda_H^0(t-d_E,a-d_E,\zeta)Z_H^0+\gamma_L Z_H^1+\mu_H W(\zeta)\mathbf{I}(a=0)-\mu_H Z_H^0 \\
\frac{\partial Z_H^k}{\partial t}+\frac{\partial Z_H^k}{\partial a}+\frac{\partial Z_H^k}{\partial \zeta} &= -\lambda_H^0(t-d_E,a-d_E,\zeta)Z_H^k+\lambda_H^0(t-d_E,a-d_E,\zeta)Z_H^{k-1}-\gamma_L k Z_H^k+\gamma_L(k+1)Z_H^{k+1}-\mu_H Z_H^k \\
\frac{\partial Z_H^K}{\partial t}+\frac{\partial Z_H^K}{\partial a}+\frac{\partial Z_H^K}{\partial \zeta} &= +\lambda_H^0(t-d_E,a-d_E,\zeta)Z_H^{K-1}-\gamma_L K Z_H^K-\mu_H Z_H^K
\end{aligned} \tag{8}$$

Note that $\mathbf{I}(a=0)$ is an indicator function ensuring that all individuals are born with age zero. The notation in supplementary equation (8) implies that Z_H^K depends on time, age and heterogeneity. However, since an individual is not assumed to change levels of heterogeneity over their lifetime, in practice we have $\frac{\partial Z_H^K}{\partial \zeta}=0$.

An individual in compartment Z_H^K will experience relapses at rate kf to give a total force of blood-stage infection of:

$$\lambda_H^k(t-d_E,a,\zeta)=\lambda_H^0(t-d_E,a,\zeta)+kf \tag{9}$$

1.5. Acquisition of immunity

In the model diagram represented in Supplementary Figure 1, a number of the transitions between compartments are depicted as being dependent on levels of anti-parasite immunity (A_P) and clinical immunity (A_C). In particular, anti-parasite immunity is assumed to have two effects: (i) reducing the probability that a blood-stage infection will achieve sufficiently high density to be detectable by light microscopy (ϕ_{LM}); and (ii) increasing the rate at which low density infections are cleared (r_{PCR}). Clinical immunity is assumed to reduce the probability that a high-density blood-stage infection (detectable by microscopy) will progress to cause a symptomatic episode of clinical malaria (ϕ_D).

Modelling the acquisition of immunity

The acquisition of both anti-parasite and clinical immunity is assumed to be age and exposure dependent. It is further assumed that both primary infections and relapses contribute to the acquisition of immunity. We follow an approach taken in previous work modelling the acquisition of immunity to *P. falciparum*[7], and assume that each new infection boosts immunity, but that after each immune boost there is a refractory period of duration u during which immunity cannot be further boosted. Under this assumption, it can be shown that if an individual is subject to a force of infection λ with refractory period u, then their immunity is boosted at rate $\frac{\lambda}{\lambda u+1}$. The acquisition of anti-parasite immunity, and its dependence on the number of batches of hypnozoites, can be described by the following partial differential equations:

$$
\begin{aligned}
\frac{\partial A_P^0}{\partial t}+\frac{\partial A_P^0}{\partial a}+\frac{\partial A_P^0}{\partial \zeta} &= -\lambda_H^0\left(t-d_E,a-d_E,\zeta\right)A_P^0 \\
&\quad -r_{\text{par}}A_P^0+\gamma_L A_P^1-\mu_H A_P^0 \\
\frac{\partial A_P^k}{\partial t}+\frac{\partial A_P^k}{\partial a}+\frac{\partial A_P^k}{\partial \zeta} &= \frac{\lambda_H^k\left(t-d_E,a-d_E,\zeta\right)}{\lambda_H^k\left(t-d_E,a-d_E,\zeta\right)u_{\text{par}}+1}-\lambda_H^0\left(t-d_E,a-d_E,\zeta\right)A_P^k+\lambda_H^0\left(t-d_E,a-d_E,\zeta\right)A_P^{k-1} \\
&\quad -r_{\text{par}}A_P^k-\gamma_L kA_P^k+\gamma_L\left(k+1\right)A_P^{k+1}-\mu_H A_P^k \\
\frac{\partial A_P^K}{\partial t}+\frac{\partial A_P^K}{\partial a}+\frac{\partial A_P^K}{\partial \zeta} &= \frac{\lambda_H^K\left(t-d_E,a-d_E,\zeta\right)}{\lambda_H^K\left(t-d_E,a-d_E,\zeta\right)u_{\text{par}}+1}+\lambda_H^0\left(t-d_E,a-d_E,\zeta\right)A_P^{K-1} \\
&\quad -r_{\text{par}}A_P^K-\gamma_L KA_P^K-\mu_H A_P^K
\end{aligned}
\tag{10}
$$

And similarly, the acquisition of clinical immunity can be described by the following system of partial differential equations:

$$
\begin{aligned}
\frac{\partial A_C^0}{\partial t}+\frac{\partial A_C^0}{\partial a}+\frac{\partial A_C^0}{\partial \zeta} &= -\lambda_H^0\left(t-d_E,a-d_E,\zeta\right)A_C^0 \\
&\quad -r_C A_C^0+\gamma_L A_C^1-\mu_H A_C^0 \\
\frac{\partial A_C^k}{\partial t}+\frac{\partial A_C^k}{\partial a}+\frac{\partial A_C^k}{\partial \zeta} &= \frac{\lambda_H^k\left(t-d_E,a-d_E,\zeta\right)}{\lambda_H^k\left(t-d_E,a-d_E,\zeta\right)u_C+1}-\lambda_H^0\left(t-d_E,a-d_E,\zeta\right)A_C^k+\lambda_H^0\left(t-d_E,a-d_E,\zeta\right)A_C^{k-1} \\
&\quad -r_C A_C^k-\gamma_L kA_C^k+\gamma_L\left(k+1\right)A_C^{k+1}-\mu_H A_C^k \\
\frac{\partial A_C^K}{\partial t}+\frac{\partial A_C^K}{\partial a}+\frac{\partial A_C^K}{\partial \zeta} &= \frac{\lambda_H^K\left(t-d_E,a-d_E,\zeta\right)}{\lambda_H^K\left(t-d_E,a-d_E,\zeta\right)u_C+1}+\lambda_H^0\left(t-d_E,a-d_E,\zeta\right)A_C^{K-1} \\
&\quad -r_C A_C^K-\gamma_L KA_C^K-\mu_H A_C^K
\end{aligned}
\tag{11}
$$

Supplementary equations (10) and (11) describe the age and exposure dependent acquisition of immunity. Another key source of immunity in young children is through maternal acquisition. We assume that a new-born infant will acquire a fraction P_{mat} of their mother's anti-parasite and clinical immunity. This immunity will then decay exponentially over time at rate $1/d_{mat}$. The levels of maternally-acquired immunity can therefore be described by the following equations:

$$A_{P,\text{mat}}\left(t,a,\zeta\right)=P_{\text{mat}}A_{P}^{*}\left(t-a,20,\zeta\right)e^{-\frac{a}{d_{\text{mat}}}}$$
$$A_{C,\text{mat}}\left(t,a,\zeta\right)=P_{\text{mat}}A_{C}^{*}\left(t-a,20,\zeta\right)e^{-\frac{a}{d_{\text{mat}}}} \quad (12)$$

The expressions $A_{P}^{*}\left(t-a,20,\zeta\right)$ and $A_{C}^{*}\left(t-a,20,\zeta\right)$ denote the level of immunity of a 20 year old woman – a representative age for women of child bearing age. * denotes that immunity levels have been averaged over the range of numbers of hypnozoite batches that it is possible for a woman to have:

$$A_{P}^{*}\left(t-a,20,\zeta\right)=\sum_{k=1}^{K}Z_{H}^{K}\left(t-a,20,\zeta\right)A_{P}^{k}\left(t-a,20,\zeta\right)$$
$$A_{C}^{*}\left(t-a,20,\zeta\right)=\sum_{k=1}^{K}Z_{H}^{K}\left(t-a,20,\zeta\right)A_{C}^{k}\left(t-a,20,\zeta\right) \quad (13)$$

Modelling the effects of immunity

The equations above describe how the acquisition and waning of immune levels can be modelled. Next we need to model how these levels of immunity affect clinically and epidemiologically relevant outcomes such as the probability that an infection becomes detectable by light-microscopy (ϕ_{LM}), the probability that an infection detectable by light-microscopy progresses to cause an episode of symptomatic malaria (ϕ_{D}), and the duration of a blood-stage infection $d_{PCR}=1/r_{PCR}$. This is done through the use of dose-response curves using flexible Hill functions:

$$\phi_{LM}^{k}\left(t,a,\zeta\right)=\phi_{LM,\min}+\left(\phi_{LM,\max}-\phi_{LM,\min}\right)\frac{1}{1+\left(\frac{A_{P}^{k}\left(t,a,\zeta\right)+A_{P,\text{mat}}\left(t,a,\zeta\right)}{A_{LM,50\%}}\right)^{\kappa_{LM}}}$$
$$\phi_{D}^{k}\left(t,a,\zeta\right)=\phi_{D,\min}+\left(\phi_{D,\max}-\phi_{D,\min}\right)\frac{1}{1+\left(\frac{A_{C}^{k}\left(t,a,\zeta\right)+A_{C,\text{mat}}\left(t,a,\zeta\right)}{A_{D,50\%}}\right)^{\kappa_{D}}} \quad (14)$$
$$d_{PCR}^{k}\left(t,a,\zeta\right)=d_{PCR,\min}+\left(d_{PCR,\max}-d_{PCR,\min}\right)\frac{1}{1+\left(\frac{A_{P}^{k}\left(t,a,\zeta\right)+A_{P,\text{mat}}\left(t,a,\zeta\right)}{A_{PCR,50\%}}\right)^{\kappa_{PCR}}}$$

1.6. Compartmental model of *P. vivax* transmission dynamics in humans

The sections above describe the dynamics of blood-stage *P. vivax* infection in humans (1.1), age structure (1.2), heterogeneity in exposure (1.3), relapses (1.4), and the acquisition of immunity (1.5). In supplementary equation (15) all of these components are combined to produce a compartmental model of *P. vivax* transmission dynamics in humans:

$$
\begin{aligned}
\frac{\partial S^k}{\partial t}+\frac{\partial S^k}{\partial a}+\frac{\partial S^k}{\partial \zeta} =& -\underbrace{\lambda_H^0\left(t-d_E,a-d_E,\zeta\right)S^k}_{\text{new infection}} - \underbrace{fkS^k}_{\text{relapse}} + \underbrace{r_{PCR}^k I_{PCR}^k}_{\text{recovery}} + \underbrace{r_P P^k}_{\text{end prophylaxis}} \\
& \underbrace{-\gamma_L k S^k + \gamma_L\left(k+1\right)S^{k+1}}_{\text{liver clearance}} - \underbrace{\mu_H S^k}_{\text{death}} + \underbrace{\mu_H W\left(\zeta\right)\mathbf{I}\left(a=0\right)}_{\text{birth}} \\
\frac{\partial I_{PCR}^k}{\partial t}+\frac{\partial I_{PCR}^k}{\partial a}+\frac{\partial I_{PCR}^k}{\partial \zeta} =& -\underbrace{\lambda_H^0\left(t-d_E,a-d_E,\zeta\right)I_{PCR}^k}_{\text{new infection}} - \underbrace{fkI_{PCR}^k}_{\text{relapse}} - \underbrace{r_{PCR}^k I_{PCR}^k}_{\text{recovery}} + \underbrace{r_{LM} I_{LM}^k}_{\text{recovery}} \\
& + \underbrace{\lambda_H^0\left(t-d_E,a-d_E,\zeta\right)\left(1-\phi_{LM}^{k-1}\right)\left(S^{k-1}+I_{PCR}^{k-1}\right)}_{\text{new PCR infection (mosquito bite)}} + \underbrace{fk\left(1-\phi_{LM}^k\right)\left(S^k+I_{PCR}^k\right)}_{\text{new PCR infection (relapse)}} \\
& \underbrace{-\gamma_L k I_{PCR}^k + \gamma_L\left(k+1\right)I_{PCR}^{k+1}}_{\text{liver clearance}} - \underbrace{\mu_H I_{PCR}^k}_{\text{death}} \\
\frac{\partial I_{LM}^k}{\partial t}+\frac{\partial I_{LM}^k}{\partial a}+\frac{\partial I_{LM}^k}{\partial \zeta} =& -\underbrace{\lambda_H^0\left(t-d_E,a-d_E,\zeta\right)I_{LM}^k}_{\text{new infection}} - \underbrace{fkI_{LM}^k}_{\text{relapse}} - \underbrace{r_{LM} I_{LM}^k}_{\text{recovery}} + \underbrace{r_D I_D^k}_{\text{recovery}} \\
& + \underbrace{\lambda_H^0\left(t-d_E,a-d_E,\zeta\right)\left(1-\phi_D^{k-1}\right)\left(\phi_{LM}^{k-1}S^{k-1}+\phi_{LM}^{k-1}I_{PCR}^{k-1}+I_{LM}^{k-1}\right)}_{\text{new LM infection (mosquito bite)}} \\
& + \underbrace{fk\left(1-\phi_D^k\right)\left(\phi_{LM}^k S^k+\phi_{LM}^k I_{PCR}^k+I_{LM}^k\right)}_{\text{new LM infection (relapse)}} \\
& \underbrace{-\gamma_L k I_{LM}^k + \gamma_L\left(k+1\right)I_{LM}^{k+1}}_{\text{liver clearance}} - \underbrace{\mu_H I_{LM}^k}_{\text{death}} \\
\frac{\partial I_D^k}{\partial t}+\frac{\partial I_D^k}{\partial a}+\frac{\partial I_D^k}{\partial \zeta} =& -\underbrace{\lambda_H^0\left(t-d_E,a-d_E,\zeta\right)I_D^k}_{\text{new infection}} + \underbrace{\lambda_H^0\left(t-d_E,a-d_E,\zeta\right)I_D^{k-1}}_{\text{new infection}} - \underbrace{r_D I_D^k}_{\text{recovery}} \\
& + \underbrace{\lambda_H^0\left(t-d_E,a-d_E,\zeta\right)\phi_D^{k-1}\left(1-\chi_T\right)\left(\phi_{LM}^{k-1}S^{k-1}+\phi_{LM}^{k-1}I_{PCR}^{k-1}+I_{LM}^{k-1}\right)}_{\text{new untreated clinical episode (mosquito bite)}} \\
& + \underbrace{fk\phi_D^k\left(1-\chi_T\right)\left(\phi_{LM}^k S^k+\phi_{LM}^k I_{PCR}^k+I_{LM}^k\right)}_{\text{new untreated clinical episode (relapse)}} \\
& \underbrace{-\gamma_L k I_D^k + \gamma_L\left(k+1\right)I_D^{k+1}}_{\text{liver clearance}} - \underbrace{\mu_H I_D^k}_{\text{death}} \\
\frac{\partial T^k}{\partial t}+\frac{\partial T^k}{\partial a}+\frac{\partial T^k}{\partial \zeta} =& -\underbrace{\lambda_H^0\left(t-d_E,a-d_E,\zeta\right)T^k}_{\text{new infection}} + \underbrace{\lambda_H^0\left(t-d_E,a-d_E,\zeta\right)T^{k-1}}_{\text{new infection}} - \underbrace{r_T T^k}_{\text{clear parasites}} \\
& + \underbrace{\lambda_H^0\left(t-d_E,a-d_E,\zeta\right)\phi_D^{k-1}\chi_T\left(\phi_{LM}^{k-1}S^{k-1}+\phi_{LM}^{k-1}I_{PCR}^{k-1}+I_{LM}^{k-1}\right)}_{\text{new treated clinical episode (mosquito bite)}} \\
& + \underbrace{fk\phi_D^k\chi_T\left(\phi_{LM}^k S^k+\phi_{LM}^k I_{PCR}^k+I_{LM}^k\right)}_{\text{new treated clinical episode (relapse)}} \\
& \underbrace{-\gamma_L k T^k + \gamma_L\left(k+1\right)T^{k+1}}_{\text{liver clearance}} - \underbrace{\mu_H T^k}_{\text{death}} \\
\frac{\partial P^k}{\partial t}+\frac{\partial P^k}{\partial a}+\frac{\partial P^k}{\partial \zeta} =& -\underbrace{\lambda_H^0\left(t-d_E,a-d_E,\zeta\right)P^k}_{\text{new infection}} + \underbrace{\lambda_H^0\left(t-d_E,a-d_E,\zeta\right)P^{k-1}}_{\text{new infection}} + \underbrace{r_T T^k}_{\text{clear parasites}} - \underbrace{r_P P^k}_{\text{end prophylaxis}} \\
& \underbrace{-\gamma_L k P^k + \gamma_L\left(k+1\right)P^{k+1}}_{\text{liver clearance}} - \underbrace{\mu_H P^k}_{\text{death}}
\end{aligned}
\tag{15}
$$

1.7. Mosquito component of model

Sections (1.1) to (1.6) describe *P. vivax* transmission dynamics in humans. Here we provide a mathematical description of *Anopheles* population dynamics, and *P. vivax* infection within mosquitoes. There are numerous important malaria vectors in Papua New Guinea. We consider the three most important species: *An. farauti s. s.*, *An. koliensis*, and *An. punctulatus*[12]. The superscript *v* is used throughout to denote different vector species. The force of infection on mosquitoes of species *v* is obtained by integrating across the entire human population:

$$\lambda_M^v(t) = \alpha^v \int_\zeta \int_a \zeta X(a)\left(c_{\mathrm{PCR}} I_{\mathrm{PCR}}(t,a,\zeta) + c_{LM} I_{LM}(t,a,\zeta) + c_D I_D(t,a,\zeta) + c_{\mathrm{T}} I_{\mathrm{T}}(t,a,\zeta)\right) da\, d\zeta \quad (16)$$

The dynamics of mosquito populations accounting for density-dependent competition in larval breeding sites are described in supplementary equation (17).

If mosquitoes are born at a constant rate β^v, the dynamics of *P. vivax* infection in mosquitoes can be described by the following equations:

$$\begin{aligned}
\frac{dL_E^v}{dt} &= \beta^v m^v - \mu_E^{0,v}\left(1 + \frac{L_E^v + L_L^v}{K^v(t)}\right) L_E^v - \frac{L_E^v}{d_E^v} \\
\frac{dL_L^v}{dt} &= \frac{L_E^v}{d_E^v} - \mu_L^{0,v}\left(1 + \gamma^v \frac{L_E^v + L_L^v}{K^v(t)}\right) L_L^v - \frac{L_L^v}{d_L^v} \\
\frac{dL_P^v}{dt} &= \frac{L_P^v}{d_L^v} - \mu_P^v L_P^v - \frac{L_P^v}{d_P^v} \\
\frac{dS_M^v}{dt} &= \frac{1}{2}\frac{L_P^v}{d_P^v} - \lambda_M^v(t) S_M^v - \mu_M^v S_M^v \\
\frac{dE_M^v}{dt} &= \lambda_M^v(t) S_M^v - \lambda_M^v(t-\tau_M^v) e^{-\mu_M^v \tau_M^v} S_M^v(t-\tau_M^v) - \mu_M^v E_M^v \\
\frac{dI_M^v}{dt} &= \lambda_M^v(t-\tau_M^v) e^{-\mu_M^v \tau_M^v} S_M^v(t-\tau_M^v) - \mu_M^v I_M^v \\
m^v &= S_M^v + E_M^v + I_M^v
\end{aligned} \quad (17)$$

S_M^v denotes susceptible adult female mosquitoes without any *P. vivax* infection. E_M^v denotes adult female mosquitoes who have been exposed to *P. vivax* following a blood-meal from an infectious human, and are thus oocyst positive but not yet infectious to humans. I_M^v denotes mosquitoes who are sporozoite positive and can transmit *P. vivax* onwards to humans. m^v denotes the total number of adult female mosquitoes of species *v* per human. Supplementary equation (17) implies that all mosquitoes die at constant rate μ_M^v independently of *P. vivax* infection status. τ_M^v is the duration of sporogony: the time required after an infectious blood meal for oocysts to develop within a mosquito and allow mosquitoes to become sporozoite positive and infect humans.

It is assumed that all adult female mosquitoes oviposit at rate β^v giving rise to early instar larvae $\left(L_E^v\right)$, which subsequently develop into late instar larvae $\left(L_L^v\right)$ and pupae $\left(L_P^v\right)$. The size of *Anopheles* populations is regulated by density-dependent competition of larvae in larval breeding site with carry capacity K^v[13]. Larval carrying capacity K^v will depend on many factors, but the primary driver is typically rainfall[13]. Supplementary equation (18) describes how seasonal variation in carrying capacity can be modelled:

$$K^{v}(t)=K_0^{v}\left(c_{\text{dry}}^{v}+\left(1-c_{\text{dry}}^{v}\right)\frac{\pi}{B\left(\frac{1}{2},\kappa^{v}+\frac{1}{2}\right)}\left(\frac{1+\cos\left(\frac{2\pi\left(t-t_{\text{peak}}^{v}\right)}{365}\right)}{2}\right)^{\kappa^{v}}\right) \tag{18}$$

In supplementary equation (18) K_0^{v} denotes the carrying capacity averaged over a season; c_{dry}^{v} represents the carrying capacity in the dry season relative to the peak of the wet season; κ^v is a shape parameter describing the degree of seasonality; θ^v is an offset parameter for the difference in time between the peak and the start of the year; and *B*() is the Beta function which is utilised to ensure appropriate normalization.

Finally, with an expression for the numbers of *P. vivax* infectious mosquitoes, we can write down an expression for the force of infection on humans:

$$\lambda_H^0\left(t,a,\zeta\right)=X\left(a\right)\zeta b\sum_{v}\alpha^{v}I_M^{v}\left(t\right) \tag{19}$$

where *b* is the probability that a bite from a *P. vivax* infectious mosquito results in a blood-stage infection.

1.8. Model likelihood and parameter estimation for human component

It is not computationally feasible to simulate a continuous partial differential equation model of the complexity shown in supplementary equation (15). This difficulty can be overcome by discretising age and heterogeneity into sub compartments, thus reducing the partial differential equation model to an ordinary differential equation model. This model can then be solved at equilibrium using methods from linear algebra, conditional upon a fixed non-seasonally varying entomological inoculation rate (EIR). A fixed EIR gives a fixed force of infection due to mosquito bites λ_H^0.

Expressions for the equilibrium values of *P. vivax* blood-stage prevalence by PCR (*Pv*PR$_{\text{PCR}}$) and light-microscopy (*Pv*PR$_{\text{LM}}$), and the prevalence of clinical episodes can be obtained as follows:

$$\begin{aligned} Pv\text{PR}_{\text{PCR}}(a) &= \frac{1}{W(a)}\int W(\zeta)\sum_k \left(I_{\text{PCR}}^k(\infty,a,\zeta) + I_{\text{LM}}^k(\infty,a,\zeta) + I_D^k(\infty,a,\zeta) + T^k(\infty,a,\zeta)\right) d\zeta \\ Pv\text{PR}_{\text{LM}}(a) &= \frac{1}{W(a)}\int W(\zeta)\sum_k \left(I_{\text{LM}}^k(\infty,a,\zeta) + I_D^k(\infty,a,\zeta) + T^k(\infty,a,\zeta)\right) d\zeta \\ Pv\text{PR}_{\text{D}}(a) &= \frac{1}{W(a)}\int W(\zeta)\sum_k \left(I_D^k(\infty,a,\zeta) + T^k(\infty,a,\zeta)\right) d\zeta \end{aligned} \tag{20}$$

Note that ∞ denotes the equilibrium values from supplementary equation (15). *Pv*PR$_{\text{D}}$(*a*) denotes the expected proportion of individuals of age *a* currently undergoing a clinical episode of *P. vivax*. This does not precisely match the epidemiological definition for the recording of episodes of clinical malaria in cross-sectional surveys, which was based on whether an individual experienced an episode in the last 48 hours (2 days). To adjust for this we can use the following formula.

$$P_D(a) = 1 - \left(1 - Pv\text{PR}_{\text{D}}(a)\right) e^{-Pv\text{PR}_{\text{D}}(a)\frac{(2\text{ days})r_D}{1-r_D}} \tag{21}$$

The likelihood that the model predicted prevalences in supplementary equations (20) and (21) fitted data from cross-sectional surveys was calculated using a Binomial likelihood. In particular, for a cross-sectional survey with *N* participants each with age a^n, let J_{PCR}^n denote PCR detectable infection ($J_{\text{PCR}}^n = 1$ if infected, $J_{\text{PCR}}^n = 0$ otherwise), let J_{LM}^n denote light-microscopy detectable infection, and let J_{D}^n denote the presence of a clinical episode in the last 48 hours. The likelihood that the parameters described the model $(\boldsymbol{\theta})$ fit the cross-sectional data $(\mathbf{D}_{\text{CS}})$ is given by

$$L(\boldsymbol{\theta}|\mathbf{D}_{\text{CS}}) = \prod_{n=1}^{N} \begin{pmatrix} \left[\left(Pv\text{PR}_{\text{PCR}}(a^n)\right)^{J_{\text{PCR}}^n} \left(1 - Pv\text{PR}_{\text{PCR}}(a^n)\right)^{1-J_{\text{PCR}}^n} \right] \\ \left[\left(Pv\text{PR}_{\text{LM}}(a^n)\right)^{J_{\text{LM}}^n} \left(1 - Pv\text{PR}_{\text{LM}}(a^n)\right)^{1-J_{\text{LM}}^n} \right] \\ \left[\left(P_{\text{D}}(a^n)\right)^{J_{\text{D}}^n} \left(1 - P_{\text{D}}(a^n)\right)^{1-J_{\text{D}}^n} \right] \end{pmatrix} \tag{22}$$

Supplementary equation (22) denotes the case for a cross-section where data is available on the presence of parasites detectable by PCR or light-microscopy and clinical cases of *P. vivax*. In some cross-sectional surveys, not all of these data types are present, in which case they don't contribute to the likelihood.

In order to fit to data on time to event from longitudinal studies, we must calculate expressions for the age and heterogeneity dependent force of infection at equilibrium. In the absence of relapses, we denote this to be $\lambda_H^0(\infty,a,\zeta)$. The contribution of relapses to the force of blood-stage infection at equilibrium can be accounted for by summing over the equilibrium distribution for the number of hypnozoite batches

$$\lambda_H^*(\infty,a,\zeta)=\lambda_H^0(\infty,a,\zeta)+\sum_{k=1}^{K}H^k(\infty,a,\zeta)kf \quad (23)$$

For a longitudinal survey with *N* participants each with age a^n, let J_{PCR}^n denote the presence of PCR detectable infection during follow-up ($J_{\mathrm{PCR}}^n=1$ if infected, $J_{\mathrm{PCR}}^n=0$ otherwise), let J_{LM}^n denote light-microscopy detectable infection, and let J_{D}^n denote a clinical episode. Let T_{PCR}^n denote the time of first PCR detectable infection during follow-up, or if there is no PCR detectable infection let T_{PCR}^n denote the time for the end of follow-up (censoring time). Let T_{LM}^n and T_{D}^n be similarly defined. The likelihood that the parameters described the model $(\boldsymbol{\theta})$ fit the longitudinal data $(\mathbf{D}_{\mathrm{L}})$ can be calculated using methods from survival analysis as follows:

$$L(\boldsymbol{\theta}|\mathbf{D}_{\mathrm{L}})=\prod_{n=1}^{N}\left(\begin{array}{l}\left[\int_0^\infty\left(\lambda_H^*(\infty,a^n,\zeta)^{J_{\mathrm{PCR}}^n}e^{-\lambda_H^*(\infty,a^n,\zeta)T_{\mathrm{PCR}}^n}\right)W(\zeta)d\zeta\right]\\ \left[\int_0^\infty\left(\left(\lambda_H^*(\infty,a^n,\zeta)\phi_{\mathrm{LM}}^*(\infty,a^n,\zeta)\right)^{J_{\mathrm{LM}}^n}e^{-\lambda_H^*(\infty,a^n,\zeta)\phi_{\mathrm{LM}}^*(\infty,a^n,\zeta)T_{\mathrm{LM}}^n}\right)W(\zeta)d\zeta\right]\\ \left[\int_0^\infty\left(\left(\lambda_H^*(\infty,a^n,\zeta)\phi_{\mathrm{LM}}^*(\infty,a^n,\zeta)\phi_{\mathrm{D}}^*(\infty,a^n,\zeta)\upsilon_{\mathrm{ACD}}\right)^{J_{\mathrm{D}}^n}e^{-\lambda_H^*(\infty,a^n,\zeta)\phi_{\mathrm{LM}}^*(\infty,a^n,\zeta)\phi_{\mathrm{D}}^*(\infty,a^n,\zeta)\upsilon_{\mathrm{ACD}}T_{\mathrm{D}}^n}\right)W(\zeta)d\zeta\right]\end{array}\right) \quad (24)$$

Note that individual-level heterogeneity in exposure to mosquito bites (which is assumed to follow a log-Normal distribution as described in supplementary equation (5)) has been integrated out. $\upsilon_{\mathrm{ACD}}=0.078$ is a correction factor as defined by Battle *et al.*[14] accounting for lower levels of incidence of clinical cases due to active case detection (ACD) every 14 – 28 days. Supplementary equation (24) denotes the case for a longitudinal study where data is available on the presence of parasites detectable by PCR or light-microscopy and clinical cases of *P. vivax*. In some longitudinal studies, not all of these data types are present, in which case they don't contribute to the likelihood.

In supplementary equation (24) relapses are assumed to contribute to the force of blood-stage infection. In one longitudinal study, half of the cohort was randomised to receive directly observed high doses of primaquine to prevent relapses[15]. In order to account for this, it is sufficient to replace $\lambda_H^*(\infty,a,\zeta)$ in supplementary equation (24) with $\lambda_H^0(\infty,a,\zeta)$.

The model for the equilibrium prevalence and incidence of *P. vivax* was fitted to the data in Supplementary Table 1 using the likelihood expressions in supplementary equations (22) and (24). The model was fitted in a Bayesian framework using Markov Chain Monte Carlo (MCMC) methods. Supplementary Table 2 describes the parameters that were estimated and the assumed prior distributions. The model was fitted twice and convergence of MCMC chains was tested by ensuring that Gelman-Rubin statistics took values < 1.01 [16]. The outputted MCMC chains were also tested to ensure that the effective sample size (ESS) for each estimated parameter was greater than 500. Posterior median parameter estimates with 95% credible intervals are provided in Supplementary Table 2.

Supplementary Table 1: Data from cross-sectional and longitudinal studies used for model calibration. Age is presented as median with range. Samples were tested for parasitaemia by PCR or light microscopy, or for a clinical case of *P. vivax* if accompanied by fever in the last 48 hours. *n*/*N* denotes *n* positive out of *N* samples.

location	study period	age (years)	PCR	LM	clinical	reference
Cross-sectional data						
Ngella, Solomon Islands	2012	18 (0.5, 100)	468/3501	127/3501	15/3501	Waltmann[27]
PNG; >1500 m	2000/02	16 (0.4, 77)		32/664	5/664	Senn[28]
PNG; 1000 – 1500 m		17 (0.6, 95)		217/2835	35/2835	Senn[28]
PNG; 500 – 1000 m		19 (0.0, 87)		446/9030	93/9030	Senn[28]
PNG; 0 – 500m		22 (0.1, 99)		290/9943	109/9943	Senn[28]
Wosera, East Sepik	1991/92, 1998/99, 2001/03	17 (0.1, 80)	901/2527	368/2527	24/2527	Mueller[29]
Wosera, East Sepik	1991/92	17.4 (0.1, 87)		1207/6782		Genton[30]
Wosera, East Sepik	2001/03	17 (0.1, 99)		1639/15737		Kasehagen[31]
Madang	2006	14.2 (0.0, 72)		204/1227	22/1227	Koepfli[32]
Ilaita & Sunuhu	2006	1.7 (0.8, 3.2)	1433/2129	1092/2129	133/2129	Lin[33]
Longitudinal data						
Mugil, Madang	2004	9.3 (4.8, 14.4)	192/204	139/204	10/204	Michon[34]
Albinama, East Sepik (placebo arm)	2008/09	7.6 (4.8, 10.4)	179/257	132/257	22/257	Robinson[15]
Albinama, East Sepik (primaquine arm)	2008/09	7.5 (4.9, 10.4)	69/247	45/247	9/247	Robinson[15]
Wosera, East Sepik	1998/99	16 (0.1, 85)		686/1689		Kasehagen[35]

Supplementary Table 2: Parameters for the human component of the model. Parameters where a prior is specified were estimated by fitting to the data in Supplementary Table 1 using Bayesian methods. Priors and posteriors are presented as medians with 95% credible intervals. Prior distributions for all parameters were log-Normally distributed, with the exception of those parameters representing proportions where a Beta distribution was assumed (†).

description	parameter	prior	posterior	reference
**exposure to mosquito bites**				
age-dependent biting parameter	ρ_{age}	fixed	0.85	Carnevale[8]; Port[9]
age-dependent biting parameter	a_0	fixed	8 years	Carnevale[8]; Port[9]
variance of log of heterogeneity in biting	σ^2	1.23 (0.51, 2.45)	1.53 (1.39, 1.66)	Smith[11]
mosquito to human infection probability	b	fixed	0.25	
**human infection duration**				
latent period (sporozoite development)	d_E	fixed	10 days	Herrera[36]
maximum PCR-detectable infection (no immunity)	$d_{PCR,\max}$	70 (24.1, 139.9)	52.6 (32.4, 84.0)	Estimated
minimum PCR-detectable infection (full immunity)	$d_{PCR,\min}$	fixed	10 days	
LM-detectable infection	d_{LM}	15 (2.2, 40.0)	16.0 (14.0, 18.2)	Estimated
clinical disease (untreated)	$1/r_D$	fixed	5 days	
clinical disease (treated)	$1/r_T$	fixed	1 day	Pukrittayakamee[37]
treatment prophylaxis	$1/r_P$	drug dependent		

proportion of episodes treated	χ_T	variable		
**anti-parasite immunity**				
anti-parasite immune boosting refractory period	u_{par}	10 (6.5, 14.3)	42.4 (36.0, 49.7) days	estimated
duration of anti-parasite immunity	d_{par}	fixed	10 years	
probability of LM-detectable infection with no immunity	$\phi_{LM,\max}$	0.85 (0.61, 0.983)†	0.93 (0.83, 0.99)	estimated
probability of LM-detectable infection with full immunity	$\phi_{LM,\min}$	0.1 (0.02, 0.22) †	0.011 (0.006, 0.016)	estimated
anti-parasite immunity for 50% reduction in LM detectable infection	$A_{LM,50\%}$	40 (4, 153)	18.8 (16.5, 21.6)	estimated
shape parameter for LM-detectable infection	κ_{LM}	2.0 (0.5, 4.4)	3.37 (2.94, 3.94)	estimated
anti-parasite immunity for 50% reduction in duration of PCR-detectable infection	$A_{PCR,50\%}$	40 (4, 153)	9.9 (6.4, 15.6)	estimated
shape parameter for duration of PCR-detectable infection	κ_{PCR}	2.0 (0.5, 4.4)	3.82 (2.56, 6.24)	estimated
**clinical immunity**				
clinical immune boosting refractory period	u_{clin}	10 (6.5, 14.3)	4.33 (2.96, 6.22)	estimated
duration of clinical immunity	d_{clin}	fixed	30 years	
probability of clinical episode with no immunity	$\phi_{D,\min}$	0.1 (0.02, 0.22) †	0.006 (0.002, 0.015)	estimated
probability of clinical episode with full immunity	$\phi_{D,\min}$	0.85 (0.61, 0.983) †	0.96 (0.86, 0.995)	estimated
clinical immunity for 50% reduction in clinical episode	$A_{D,50\%}$	40 (4, 153)	24.5 (20.8, 29.1)	estimated
shape parameter for clinical episode probability	κ_D	2.0 (0.5, 4.4)	5.63 (3.75, 8.34)	estimated
**maternal immunity**				
new-born immunity relative to mother's	P_{mat}	0.5 (0.13, 0.87) †	0.31 (0.11, 0.68)	estimated
duration of maternal immunity	d_{mat}	60 (27, 105)	49.9 (23.0, 123.6)	estimated
**infectiousness to mosquitoes**				
during PCR-detectable infection	c_{PCR}	fixed	0.035	Kiattibutr[38]
during LM-detectable infection	c_{LM}	fixed	0.1	Kiattibutr[38]
during clinical disease	c_D	fixed	0.8	Kiattibutr[38]
during treated clinical disease	c_T	fixed	0.4	Kiattibutr[38]
**relapse parameters**				
relapse frequency	f	fixed	1/41 days^{-1}	White[15]
liver-stage hypnozoite clearance rate	γ_L	fixed	1/383 days^{-1}	White[15]

The mathematical model described above and the parameters presented in Supplementary Table 2 imply certain relationships between level of anti-parasite and clinical immunity, and the prevalence and incidence of *P. vivax*. Supplementary Figure 2 gives an overview of some of these relationship at a representative range of EIRS.

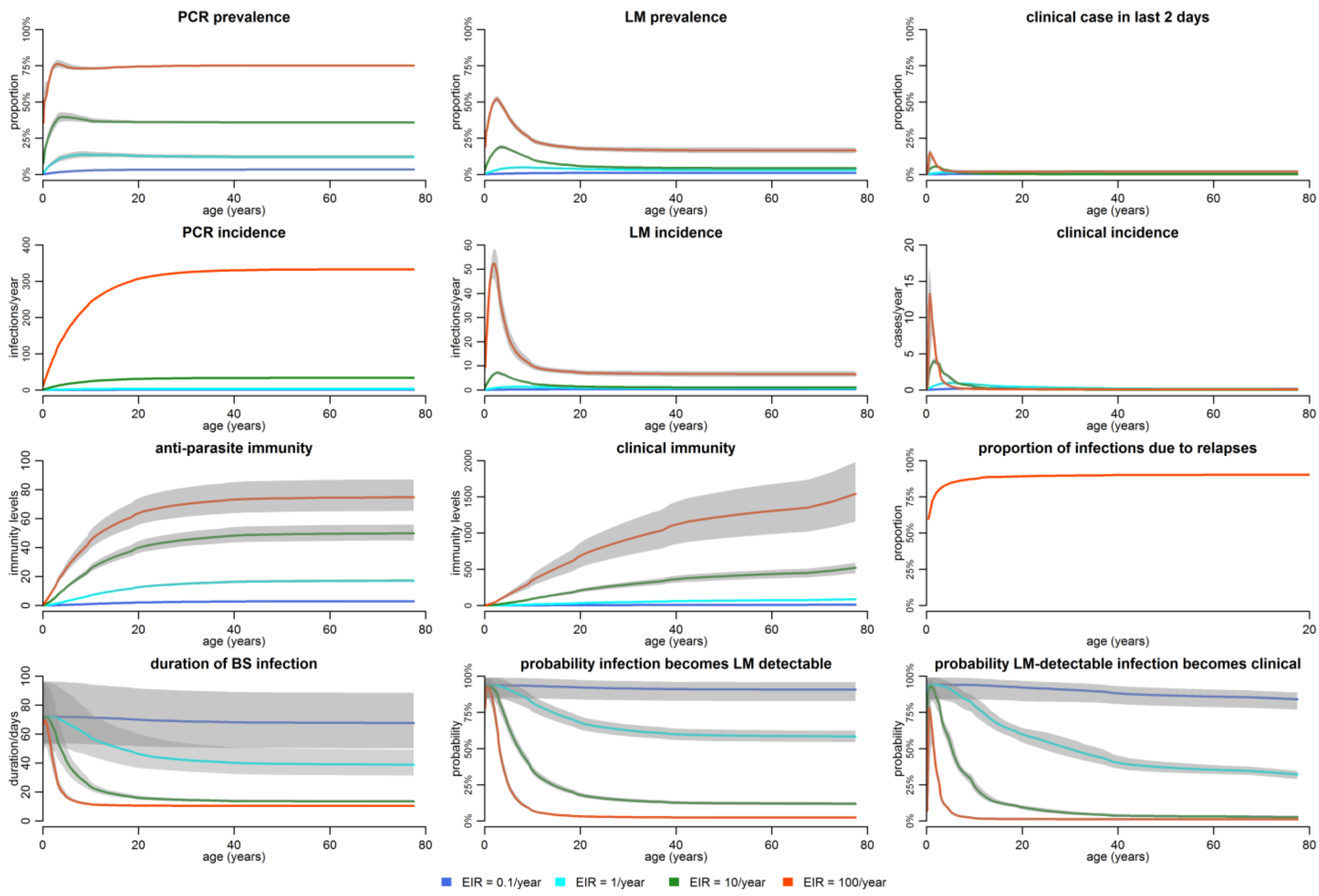

Supplementary Figure 2: Association between prevalence, incidence and levels of acquired immunity at a range of entomological inoculation rates (EIR) predicted by the model using the parameters detailed in Supplementary Table 2.

1.9. Parameters for the mosquito component of the model

In order to parameterise the mosquito component of the model, we reviewed data on entomological parameters from a wide range of studies across Papua New Guinea and the Solomon Islands (Supplementary Table 3).

Supplementary Table 3: Mosquito bionomics parameters for the three primary malaria vectors in Papua New Guinea: *An. farauti s. s.*, *An. punctulatus*, and *An. koliensis*. The table below shows parameters extracted from the referenced publications for each of the three species. The values shown in the grey shaded region denote the values assumed in the model.

**description**	**parameter**	***mosquito species***			**reference**
		An. farauti s. s.	***An. punctulatus***	***An. koliensis***	
mosquito life expectancy (days)	$1/\mu_M^v$	6			Clements[39]
		6	6	6	
duration of sporogony (days)	τ_M^v	8.4	8.4	8.4	Gething[40]
		8.4	8.4	8.4	
duration of gonotrophic cycle (days)	δ^v	2.5			Bugoro[25]
		2.2, 2.47			Charlwood[41]
		2.1, 3	2.9, 2.4, 3.2	2.7, 3.7	Charlwood[42]
		3	3	3	
time spent foraging (days)	τ_1^v	0.68	0.68	0.68	Killeen[43]
		0.68	0.68	0.68	
human blood index	Q_0^v	77%, 47%, 50%	74%, 65%		Keven[44]
		93%			Russell[45]
			72%		Killeen[46]
		88%	90%	79%	Hii[47]
		11%, 25%, 8%, 45%	86%, 71%, 100%, 60%, 20%, 50%, 50%75%, 26%	29%, 76%, 36%, 82%, 85%	Burkot[48]
			84%, 46%, 96%, 90%		Burkot[49]
			86%, 98%		Burkot[50]
		12%	55%	65%	Burkot[51]
		72%		83%	Charlwood[52]
		52%, 83%		58%, 95%	Charlwood[42]
		50%	77.5%	73%	
night-time biting		80%, 80%			
		24%			Russell[53]
		18%			Russell[54]
		62%	84%	85%	Reimer[26]
		35%			Bugoro[55]
		50%			Bugoro[56]
		50%	84%	85%	
indoor biting		16.5%			Thomsen[57]
		28%			Russell[53]
		35%			Russell[54]
		36%			Bugoro[55]
		55%			Bugoro[56]
		57%, 37%			Bugoro[25]
		72%	87%	95%	Hii[47]
			66%, 81%	56%	Sama[58]
		36.5%	81%	74.5%	
indoor resting			88%, 93%		Burkot[49]
		90.5%	90.5%	90.5%	

A number of entomological studies presented data on the seasonally variation of captured adult female mosquitoes. The data are presented in Supplementary Figure 3. Although there was notable seasonal variation in capture rates between villages sampled at different times, there was a clear seasonal pattern with mosquito densities tending to peak around October. The seasonally varying functional form described in supplementary equation (17) was fitted to the data with the resulting parameters presented in Supplementary Table 4.

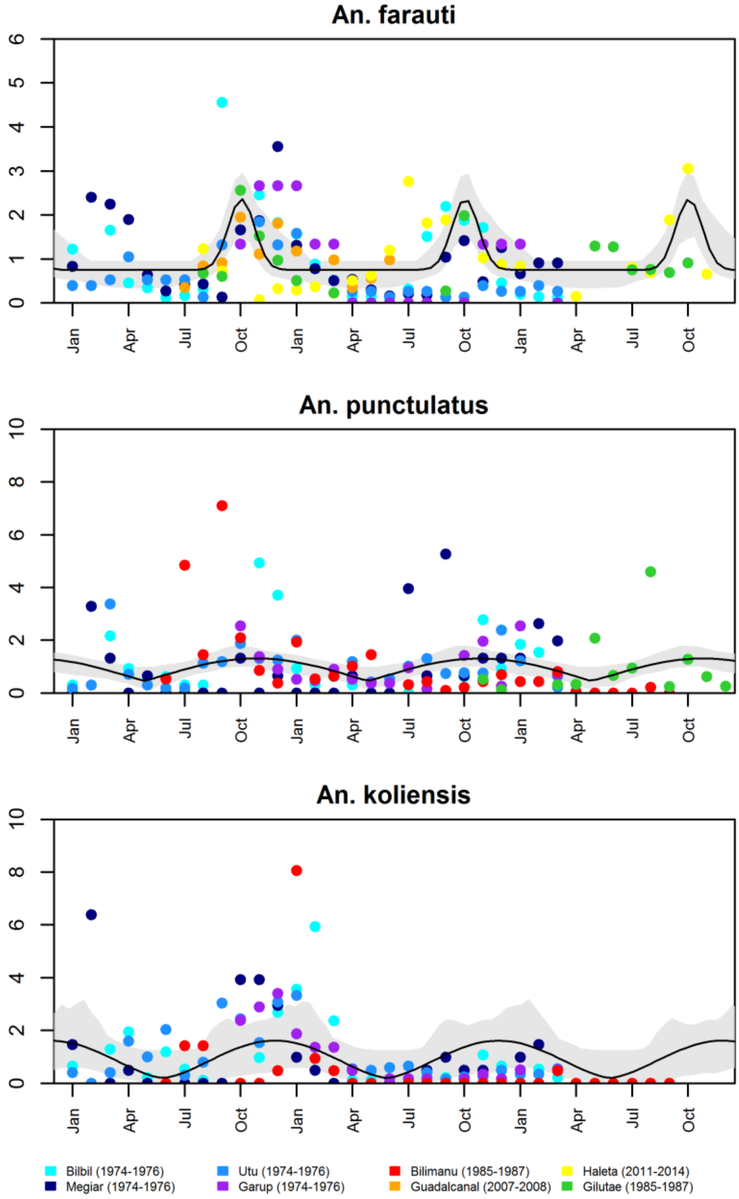

Supplementary Figure 3: Data on seasonal abundance of three species of adult female Anophelines from villages in Papua New Guinea and the Solomon Islands. Data from Bilbil, Megiar, Utu and Garup are from Afifi *et al*[63]. Data from Bilimanu are from Samarawickrema *et al*[58]. Data from Guadalcanal are from Bugoro *et al*[55]. Data from Gilutae are from Kere *et al*[64]. Data from Haleta are from Russell *et al*[54]. The black line shows the fitted seasonal form described by supplementary equation (18) and the shaded grey region shows the 95% confidence interval.

Supplementary Table 4: Mosquito seasonality parameters with 95% confidence intervals estimated from the data shown in Supplementary Figure 3.

**description**	**parameter**	***mosquito species***		
		An. farauti s. s.	***An. punctulatus***	***An. koliensis***
dry season proportion	c^{v}_{dry}	0.317 (0.18, 0.47)	0.347 (0.03, 0.56)	0.124 (0.06, 0.82)
seasonality shape parameter	κ^{v}	14.6 (1.22, 19.9)	0.52 (0.26, 16.5)	0.98 (0.10, 14.7
seasonal peak offset (days)	t^{v}_{peak}	306 (294, 335)	330 (0, 364)	360 (266, 365)

The mosquito component of the model also describes the dynamics of larval populations (supplementary equation (17)), based on a previously published model of the dynamics of the African malaria vector *An. gambiae*[13]. The parameters for this model are presented in Supplementary Table 5.

Supplementary Table 5: Parameters describing larval development and density-dependent competition from White *et al*[13]. The same parameter values are assumed for each of the three key Papua New Guinean mosquito species.

description	parameter	value
development time of early larval instars	d_E^v	6.64 (days)
development time of late larval instars	d_L^v	3.72 (days)
development time of pupae	d_P^v	0.64 (days)
per capita daily mortality rate of early instars (low density)	$\mu_E^{0,v}$	0.034 day^{-1}
per capita daily mortality rate of late instars (low density)	$\mu_L^{0,v}$	0.035 day^{-1}
per capita daily mortality rate of pupae	μ_P^v	0.25 day^{-1}
number of eggs laid per day per mosquito	β^v	21.19
effect of density dependence on late instars relative to early instars	γ^v	13.25

1.10. Individual-based model

Sections 1.1. to 1.9. describe a mathematical model of *P. vivax* transmission using the notation of compartmental differential equation models. Notable points of using compartmental models are: (i) they are deterministic and cannot produce stochastic variation; (ii) they are not well suited to modelling elimination events; and (iii) implementing combinations of interventions requires enormous increases in the numbers of modelled compartments. Any compartmental model of infectious disease transmission can also be implemented as an individual-based model[17]. Here we provide an overview of the implementation of this model as an individual-based model.

Populations of size 100,000 are initialised, with each individual being assigned:

- age drawn from the distribution in supplementary equation (2)
- factor for heterogeneity in exposure to mosquito bites from the distribution in supplementary equation (5)
- sex (50% male; 50% female)
- pregnant (possible if female and between the ages 18 and 40 years)
- G6PD status
- CYP2D6 metabolizer phenotype
- LLIN use
- *P. vivax* infection status (susceptible S; infected with PCR-detectable blood-stage parasites I_{PCR}; infected with LM-detectable blood-stage parasites I_{LM}; clinical episode D_D; under treatment T; under blood-stage prophylaxis P)
- number of batches of hypnozoites
- anti-parasite immunity A_P (accounting for maternal immunity if age less than 1 year)
- clinical immunity A_C (accounting for maternal immunity if age less than 1 year)

When individuals are initialised, the levels of *P. vivax* infection and naturally-acquired immunity in the population are set to match the equilibrium of the non-seasonal compartmental differential equation model in supplementary equation (15).

All individuals in the simulated population are updated with a time step of 1 day. Most processes occur at a constant rate. If a process occurs at a constant rate *r*, then the probability of it occurring in time step Δt is 1 – e(-r* Δt). Some processes occur after a fixed period of time (e.g. development from sporozoites to merozoites in the liver). These are implemented using a timer. Modelled process that are not directly related to *P. vivax* transmission are:

- ageing (increased by Δt every time step)
- death
- birth (scheduled so that a birth occurs after every death to ensure a balanced population)
- pregnancy (occurs at a constant rate, ends after a fixed duration of 9 months)
- loss of LLIN adherence

The process related to *P. vivax* infection are exactly the same as those implemented in the compartmental model in supplementary equation (15).

Mosquitoes are not modelled individually, and the compartmental differential equations are still used. In particular, the ODEs are solved using a 4 step Runge-Kutta algorithm with a time step of $0.1\Delta t$.

Heavily commented C++ code for implementing the individual-based model is available for download from GitHub @MWhite-InstitutPasteur.

2. Supplementary methods: Intervention models

2.1. Vector control: long lasting insecticidal nets (LLINs)

We implement an existing model of the effects of LLINs on *Anopheles* vectors[7,18]. LLINs have a number of key effects on mosquitoes: (i) they increase mosquito death rates; (ii) they increase time spent searching for a blood meal thus increasing the duration of the gonotrophic cycle; (iii) they can reduce the mosquito population size as a consequence of reduced oviposition; (iv) they change the proportion of bites taken on protected versus unprotected people; (v) they change the proportion of bites taken on humans relative to animals (the Human Blood Index). Supplementary Figure 4 provides an overview of the interactions between mosquitoes, humans and LLINs.

We denote χ_{LLIN} to be the coverage of LLINs. Although there are several metrics of LLIN coverage[19], the metric most suitable for modelling is the proportion of individuals sleeping under a net. This is typically measured in surveys via answers to the question "Did you sleep under a bed net last night?".

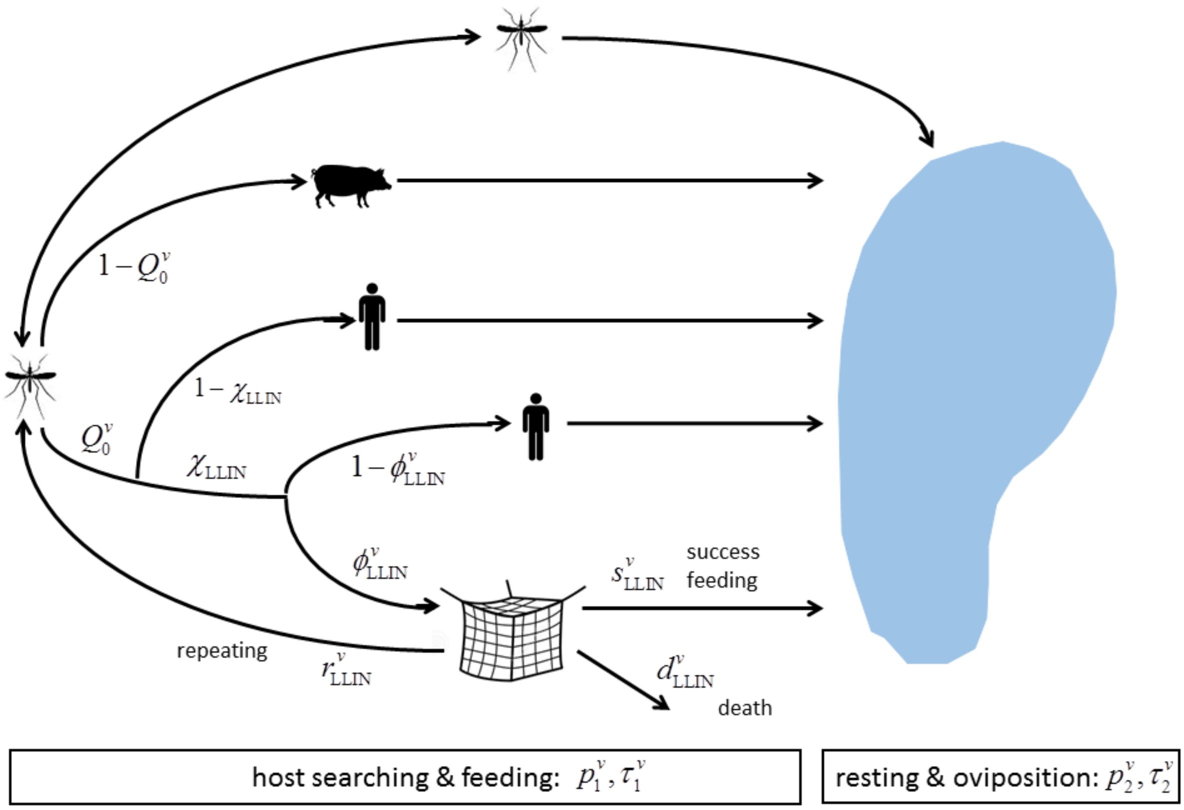

Supplementary Figure 4: Flow chart of the mosquito life cycle and its interactions with long-lasting insecticidal nets (LLINS), adapted from a model by Le Menach *et al*[18].

A mosquito will feed on humans at a rate α^v. This rate can be broken down into two terms, the average time to complete one feeding cycle $\delta^v = 1/f^v$, and the proportion of bites taken on humans Q_0^v.

$$\alpha^v = f^v Q_0^v \tag{25}$$

From the flow chart in Supplementary Figure 4, the probability that a surviving mosquito succeeds in feeding during a single attempt can be calculated to be

$$\begin{aligned} w^v &= 1 - Q_0^v + Q_0^v(1-\chi_{\text{LLIN}}) + Q_0^v\chi_{\text{LLIN}}(1-\phi_{\text{LLIN}}^v) + Q_0^v\chi_{\text{LLIN}}\phi_{\text{LLIN}}^v s_{\text{LLIN}}^v \\ w^v &= 1 - Q_0^v\chi_{\text{LLIN}}\phi_{\text{LLIN}}^v(1-s_{\text{LLIN}}^v) \end{aligned} \tag{26}$$

where χ_{LLIN} is the coverage of bed nets, ϕ^{v}_{LLIN} is the proportion of people in bed when a mosquito attempts to bite, and s^{v}_{LLIN} is the probability of a mosquito feeding successfully on a person sleeping under a bed net. The probability of a mosquito resetting and beginning a new search is

$$z^{v} = Q^{v}_{0}\chi_{\mathrm{LLIN}}\phi^{v}_{\mathrm{LLIN}}r^{v}_{\mathrm{LLIN}} \tag{27}$$

where r^{v}_{LLIN} is the probability of a mosquito being repelled by a bed net. At zero LLIN coverage the length of a feeding cycle is given by

$$\frac{1}{f^{v}} = \tau^{v}_{1}(0) + \tau^{v}_{2} \tag{28}$$

where $\tau^{v}_{1}(0)$ is the time spent searching for a blood meal, and τ^{v}_{2} is the time spent resting. Increased LLIN coverage will cause the mosquito to spend a longer time foraging for a blood meal. And hence at coverage χ_{LLIN}

$$\tau^{v}_{1}(\chi_{\mathrm{LLIN}}) = \tau^{v}_{1}(0) + z^{v}\tau^{v}_{1}(\chi_{\mathrm{LLIN}}) = \frac{\tau^{v}_{1}(0)}{1-z^{v}} \tag{29}$$

And therefore the length of a feeding cycle at coverage χ_{LLIN} is

$$\frac{1}{f^{v}(\chi_{\mathrm{LLIN}})} = \frac{\tau^{v}_{1}(0)}{1-z^{v}} + \tau^{v}_{2} \tag{30}$$

Increased LLIN coverage will also reduce the probability of a mosquito surviving the foraging stage p^{v}_{1}.

$$p^{v}_{1}(\chi_{\mathrm{LLIN}}) = p^{v}_{1}(0)[w^{v} + z^{v}p^{v}_{1}(\chi_{\mathrm{LLIN}})] = \frac{p^{v}_{1}(0)w^{v}}{1-z^{v}p^{v}_{1}(0)} \tag{31}$$

Thus the probability of a mosquito surviving one day is given by

$$p^{v}(\chi_{\mathrm{LLIN}}) = \left(p^{v}_{1}(\chi_{\mathrm{LLIN}})p^{v}_{2}\right)^{f^{v}(\chi_{\mathrm{LLIN}})} = \left(\frac{p^{v}_{1}(0)w^{v}p^{v}_{2}}{1-z^{v}p^{v}_{1}(0)}\right)^{f^{v}(\chi_{\mathrm{LLIN}})} \tag{32}$$

where p^{v}_{2} is the probability of surviving resting. The mosquito mortality can then be calculated as

$$\mu^{v}_{M}(\chi_{\mathrm{LLIN}}) = -\log p^{v}(\chi_{\mathrm{LLIN}}) \tag{33}$$

In addition to killing mosquitoes and altering their feeding behaviour, bed nets can reduce mosquito population by reducing the oviposition rate. It can be shown (White 2011) that if a female mosquito oviposits η^{v} eggs every $\delta^{v} = 1/f^{v}$ days, then the mean daily oviposition rate is

$$\beta^{v} = \eta^{v}\frac{\mu^{v}_{M}}{e^{\mu^{v}_{M}/f^{v}} - 1} \tag{34}$$

LLINs increase the death rate and increase the time spent searching for a blood meal, resulting in a decreased oviposition rate as follows

$$\beta^{v}(\chi_{\mathrm{LLIN}}) = \eta^{v}\frac{\mu^{v}_{M}(\chi_{\mathrm{LLIN}})}{e^{\mu^{v}_{M}(\chi_{\mathrm{LLIN}})/f^{v}(\chi_{\mathrm{LLIN}})} - 1} \tag{35}$$

Changing coverage and insecticide decay

The degree of protection conferred by LLINs reduces over time for two key reasons: (i) people stop using nets over the time as they age or get lost; (ii) the insecticidal activity of nets reduces over time.

Based on data on data on the age distribution of bed nets collected in Papua New Guinea[20], we assume that the average lifespan of a LLIN is T_{LLIN} = 19.5 months (1.62 years), so that coverage at time t after initial distribution is

$$\chi_{\mathrm{LLIN}}(t) = \chi_{\mathrm{LLIN}}(0)\,\mathrm{e}^{-t/T_{\mathrm{LLIN}}} \tag{36}$$

We assume that both the killing and repellency effects of LLINs wane over time at the same rate γ_{LLIN}. Even after a net's insecticidal repellency has fully waned, we assume there is residual repellency due to the barrier action of the net. At time t after distribution, the probabilities of a mosquito being repelled, dying or feeding successfully upon encounter with a net are given by

$$\begin{aligned} r_{\mathrm{LLIN}}(t) &= r_{\mathrm{LLIN},0} + \left(r_{\mathrm{LLIN}}(t) - r_{\mathrm{LLIN},0}\right)e^{-\gamma_{LLIN}t} \\ d_{\mathrm{LLIN}}(t) &= d_{\mathrm{LLIN}}(t)e^{-\gamma_{LLIN}t} \\ s_{\mathrm{LLIN}}(t) &= 1 - r_{\mathrm{LLIN}}(t) - d_{\mathrm{LLIN}}(t) \end{aligned} \tag{37}$$

The effect of LLINs on mosquito populations depends on the parameters describing a mosquito's behaviour when attempting to take a blood meal from a person sleeping under a net. In African settings, these parameters have been estimated through experimental hut trials[21]. Suitable parameter estimates are not available for Papua New Guinean malaria vectors, and thus we use values from African studies[7] (Supplementary Table 6).

Supplementary Table 6: Parameters describing mosquito behaviour upon encounter with an LLIN (Supplementary Figure 4). The same values are assumed for each of the three primary malaria vectors in Papua New Guinea.

description	parameter	value
half-life of LLINs	T_{LLIN}	1.62 (years)
half-life of pyrethroid insecticide	γ_{LLIN}	2.5 (years)
probability mosquito repelled by LLIN (full insecticide activity)	r_{LLIN}	0.6
probability mosquito repelled by LLIN (just barrier activity)	$r_{\mathrm{LLIN,0}}$	0.2
probability mosquito killed by LLIN (full insecticide activity)	d_{LLIN}	0.3
probability mosquito feeds successfully (full insecticide activity)	s_{LLIN}	0.1

2.2. First-line treatment

First-line treatment of symptomatic episodes of *P. vivax* provides clinical benefit to the individual being treated, and may also reduce population-level transmission by preventing onwards transmission to mosquitoes. A key variable for assessing the effectiveness of treatment strategies is the proportion of *P. vivax* clinical cases that receive effective treatment (χ_T).This value is very difficult and potentially impossible to measure directly, and will depend on the definition of what constitutes a clinical episode of *P. vivax*, the proportion of cases detected[14], the proportion of individuals that seek treatment, and the proportion of health facilities that have sufficient stocks of drugs[22]. Conditional upon an individual with a symptomatic episode of *P. vivax* choosing to seek treatment, they will enter a treatment pathway which can be modelled. Supplementary Figure 5 shows a treatment pathway for individuals receiving primaquine. The parameters for this pathway are provided in Supplementary Table 7.

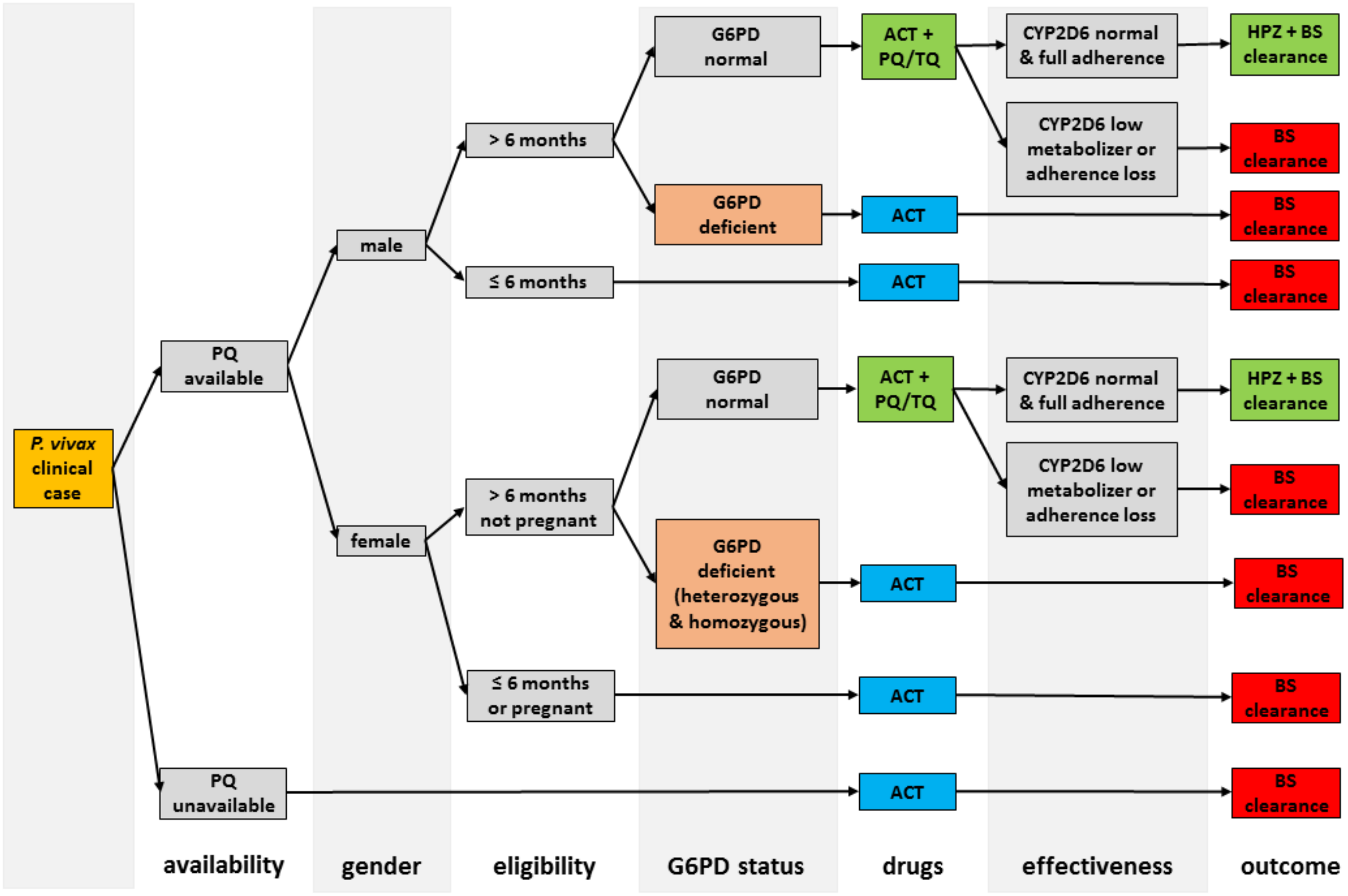

Supplementary Figure 5: Overview of pathway for first-line treatment of symptomatic episodes of *P. vivax* incorporating primaquine treatment for the clearance of hypnozoites and the prevention of relapses.

Supplementary Table 7 presents the parameters for the treatment pathway in Supplementary Figure 5. Supplementary Table 7 also presents estimates of the effectiveness of a drug regimen for clearing hypnozoites based on the treatment pathway in Supplementary Figure 5. These estimates are efficacy are conditional on drugs being in stock, and the availability of point-of-care G6PD diagnostics (not routinely available anywhere in Papua New Guinea at present). We assume the primaquine and tafenoquine cannot be given to children < 6 months age. Note that the age range for who can safely receive tafenoquine has not yet been defined.

Supplementary Table 7: Parameters for treatment and diagnosis assumptions in the treatment pathway in Supplementary Figure 5. * Primaquine and tafenoquine lose effectiveness due to the inability to provide safe treatment to infants, pregnant women and G6PD deficient individuals. Primaquine further loses effectiveness in low CYP2D6 metabolizers, and in individuals who fail to adhere to the full treatment regimen.

**description**	**parameter**	**value**	**reference**
human demographics			
average age of humans (years)	$1/\mu_H$	22.5	CIA factbook
maximum age of humans (years)	a_{max}	80	assumption
human death rate (year^{-1})	μ_H	0.044	
proportion male		50%	assumption
proportion < 6 months of age		2.2%	from equation (2)
proportion of pregnant women		4.0%	assumption
genotype & phenotype prevalence			
G6PD deficiency prevalence (males)	q_{G6PD}	7.42%	Howes[59]
G6PD deficiency prevalence (homozygous females)	$q_{\mathrm{G6PD}} * q_{\mathrm{G6PD}}$	0.55%	Howes[59]
G6PD deficiency prevalence (heterozygous females)	$2*q_{\mathrm{G6PD}}*(1- q_{\mathrm{G6PD}})$	13.7%	Howes[59]
low CYP2D6 metabolizer phenotype	q_{CYP2D6}	5.0%	assumption
treatment			
ACT blood-stage efficacy		100%	Karunajeewa[60]
ACT duration of prophylaxis (days)		14	Karunajeewa[60]
primaquine (0.25 mg/kg 14 days) effectiveness		70%	Assumption
primaquine (0.25 mg/kg 14 days) prophylaxis (days)		14	John[61]
tafenoquine (single dose) effectiveness		100%	Llanos-Cuentas[62]
tafenoquine (single dose) prophylaxis (days)		60	Llanos-Cuentas[62]
effectiveness against hypnozoites			
ACT		0%	
primaquine*		55.3%	
tafenoquine*		83.2%	

2.3. Mass drug administration

Mass drug administration (MDA) involves treating a large proportion of a population with drugs in a short period of time. In the model, MDA at coverage χ_{MDA} is implemented at a fixed time by having a proportion of individuals enter the treatment pathway in Supplementary Figure 5. Notably, we assume that an MDA campaign with primaquine or tafenoquine is implemented with testing for G6PD deficiency.

3. Supplementary Methods: *P. vivax* transmission in Papua New Guinea

We model *P. vivax* transmission in Papua New Guinea on the provincial level. The nationwide household prevalence surveys implemented by Hetzel *et al* were explicitly designed to take measurements of malaria transmission and bed net coverage from each province[23,24]. Within each province we assume freely mixing populations of humans and mosquitoes. We assume that levels of malaria transmission and access to interventions are constant throughout each province. In particular, we do not account for spatial variation in transmission intensity within each province which is known to vary substantially. Ideally we would be able to model transmission at the lower levels of Districts or Local Level Government (LLG) area. However, there is not sufficient data to meaningfully model malaria transmission at any spatial scale lower than the province level. A further assumption is that we don't model importation of people and parasites between provinces.

It is assumed that LLIN coverage increases to the levels indicated in Supplementary Table 8 in the years 2009, 2011 and 2014, and that nets have a life expectancy of T_{LLIN} = 19.5 months such that coverage reduces over time according to supplementary equation (36). Furthermore, the metric of coverage utilised is based on use – the proportion of individuals sleeping under a net during the previous night.

Supplementary Table 8: Provincial level data on *P. vivax* light microscopy prevalence and LLIN use.

	***P. vivax* microscopy prevalence**				**LLIN use**		
	sentinel survey	post-LLIN survey	household surveys				
	2009	2009	2010	2014	2009	2011	2014
Western (Fly)	0.3%	0.0%	0.3%	0.0%	40.4%	67.2%	59.9%
Gulf		0.0%	0.0%	0.0%	40.4%	67.2%	59.9%
Central		0.7%	0.3%	0.0%	40.4%	67.2%	59.9%
Milne Bay		1.5%	4.3%	0.93%	40.4%	67.2%	59.9%
Oro (Northern)		2.9%	0.4%	0.0%	40.4%	67.2%	59.9%
Southern Highlands		1.3%	0.6%	0.0%	22.7%	39.6%	37.3%
Enga		1.3%	0.0%	0.0%	22.7%	39.6%	37.3%
Western Highlands	2.4%	1.5%	0.0%	0.53%	22.7%	39.6%	37.3%
Chimbu			0.0%	0.0%	22.7%	39.6%	37.3%
Eastern Highlands		0.9%	0.0%	0.0%	22.7%	39.6%	37.3%
Morobe	3.0%	0.2%	1.2%	0.0%	47.0%	48.7%	68.2%
Madang	3.4%	2.5%	1.0%	0.22%	47.0%	48.7%	68.2%
East Sepik		0.6%	2.1%	0.43%	47.0%	48.7%	68.2%
Sandaun (West Sepik)	9.2%	0.5%	4.5%	2.17%	47.0%	48.7%	68.2%
Manus		2.7%	1.3%	0.55%	25.4%	39.9%	53.9%
New Ireland		3.6%	7.1%	1.21%	25.4%	39.9%	53.9%
East New Britain		3.2%	3.4%	2.69%	25.4%	39.9%	53.9%
West New Britain		12.6%	4.9%	1.1%	25.4%	39.9%	37.3%
Bougainville		3.8%	4.9%	0.0%	25.4%	39.9%	53.9%

There are a large number of species of *Anopheles* known to be implicated in malaria transmission throughout Papua New Guinea. We account for three most common species: *An. farauti sensu stricto* (*s. s.*), *An. punctulatus*, and *An. koliensis*. *An. farauti s. s.* is found almost exclusively in coastal areas[12], and is known to be an early evening biter, thus making it resilient to many vector control tools[25]. *An. farauti s. s.* is part of the wider species complex *An. farauti sensu lato* (*s. l.*). Although we do not attempt to model the wider species complex, we do note that *An. farauti* 4 is a potentially important vector for malaria transmission in inland regions. *An. punctulatus* and *An. koliensis* are found throughout Papua New Guinea, with a greater tendency to be reported in inland areas.

There are many entomological studies where species identification was performed on captured *Anophelines* and the relative proportion of different species reported[26]. However, these studies are restricted to a small number of well characterised study sites. We based the relative proportions of the three primary malaria vectors on the results of a large survey of *Anophelines* in Papua New Guinea[12]. In this study, helicopters were used to sample hundreds of larval breeding sites over seven years from across mainland Papua New Guinea. These survey results for the presence or absence of mosquito species were used to inform the proportions in Supplementary Table 9.

Supplementary Table 9: Estimates of the relative proportions of the three primary malaria vectors in Papua New Guinea: *An. farauti*, *An. punctulatus*, and *An. koliensis*. Estimates are based on observations by Cooper *et al*[12].

	An. farauti s. s.	*An. punctulatus*	*An. koliensis*
Western (Fly)	50%	25%	25%
Gulf	50%	25%	25%
Central	50%	25%	25%
Milne Bay	50%	50%	0%
Oro (Northern)	25%	50%	25%
Southern Highlands	50%	50%	0%
Enga	0%	50%	50%
Western Highlands	0%	50%	50%
Chimbu	0%	50%	50%
Eastern Highlands	0%	50%	50%
Morobe	25%	50%	25%
Madang	25%	50%	25%
East Sepik	25%	25%	50%
Sandaun (West Sepik)	25%	25%	50%
Manus	50%	25%	25%
New Ireland	50%	25%	25%
East New Britain	50%	25%	25%
West New Britain	50%	25%	25%
Bougainville	50%	25%	25%

4. Supplementary methods: *P. vivax* simulation model with inter-provincial travel

The provincial-level simulations presented in the main manuscript do not account for the role of travel of individuals between provinces. Movement of individuals between provinces is likely to cause challenges for attempts to control and eliminate *P. vivax* transmission, particularly if a province has eliminated transmission, but a neighbouring province hasn't. To investigate the potential impact of movement of people and parasites between provinces in Papua New Guinea, we estimated the number of inter-provincial journeys by air, land and sea. A map of Papua New Guinean roads is shown in Supplementary Figure 6. A pair of provinces are considered to be connected if they are linked by road. Neighbouring coastal provinces are considered to be connected by sea. We used local knowledge of boat routes to denote connectivity between island provinces. Using this land and sea connectivity matrix, we estimated numbers of journeys by assuming that, 20% of individuals in all provinces take one journey to another province. The numbers of journeys is therefore dependent on the population of each province (Supplementary Table 10). The estimated number of daily journeys between provinces by land and sea is shown in Supplementary Figure 7a. Maps were created using the maptools R package with shape files downloaded from http://otlet.sims.berkeley.edu/imls/world/PNG/.

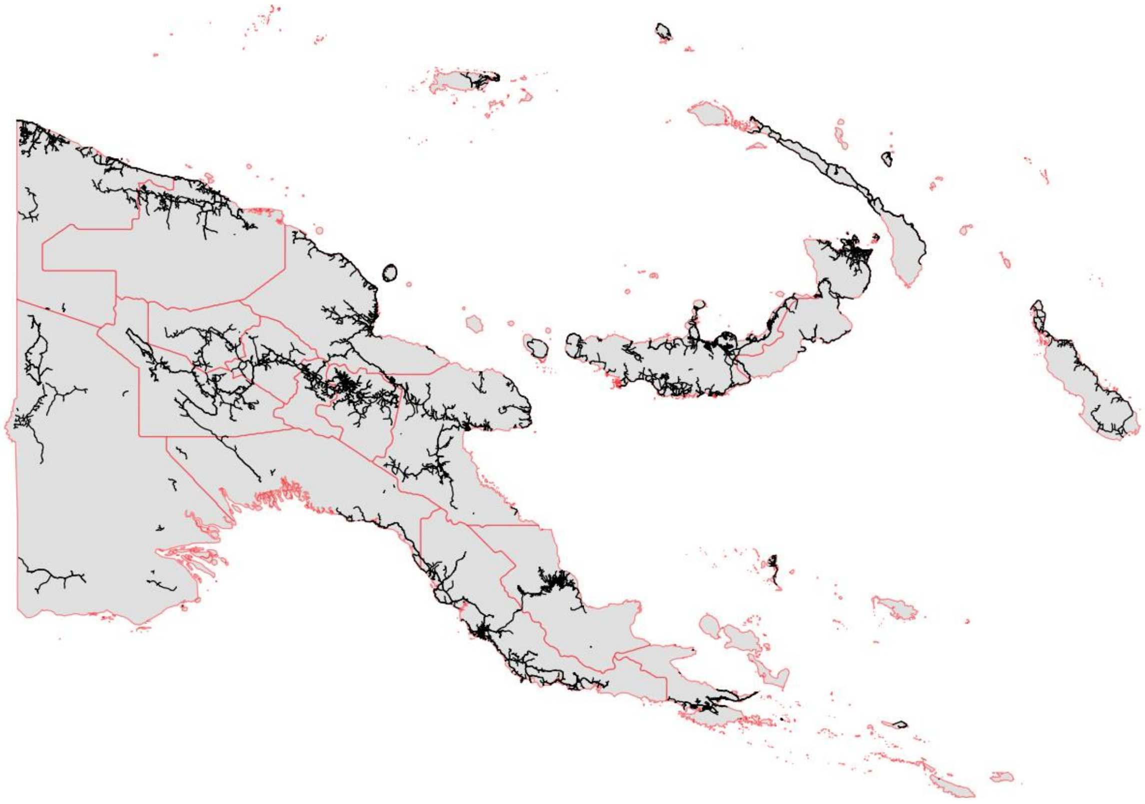

Supplementary Figure 6: Map of Papua New Guinean roads.

Supplementary Table 10: Estimated population of each province from the 2011 Papua New Guinean census. https://www.nso.gov.pg/index.php/population-and-social/migration/24-population-and-social

**Province**	**Population**
Southern Region	
Western	201,351
Gulf	158,197
Central (including National Capital District)	633,881
Milne Bay	276,512
Oro (Northern)	186,309
Islands Region	
Manus	60,485
New Ireland	194,067
East New Britain	328,369
West New Britain	264,264
Bougainville	249,358
Momase Region	
Morobe	674,810
Madang	493,906
East Sepik	450,530
Sandaun (West Sepik)	248,411
Highlands Region	
Southern Highlands (includes Hela)	759,694
Enga	432,045
Western Highlands (includes Jiwaka)	706,837
Chimbu	376,473
Eastern Highlands	579,825

A high proportion of journeys in PNG are undertaken by air. We downloaded the weekly flight schedules for Air Niugini and PNG Air, the two airlines responsible for all non-private domestic flights. By accounting for the make of each aircraft, we were able to estimate the average daily number of seats between provinces. By assuming a seat occupancy rate of 70%, we were able to estimate the average daily number of air journeys between provinces. Jacksons Airport in Port Moresby, Central Province is the key airline hub in PNG. We therefore assumed the 33% of domestic travel through this airport was for connecting flights. A common example of this sort of travel is encountered by members of our study wishing to travel between the PNG Institute of Medical Research's sites in Goroka (Eastern Highlands Province) and Yagaum (Madang Province). This is a journey of approximately 300km by road, but in occasions where the road is not passable, individual must travel by air, connecting through Port Moresby. A map of estimated daily journeys by air between provinces is shown in Supplementary Figure 7b.

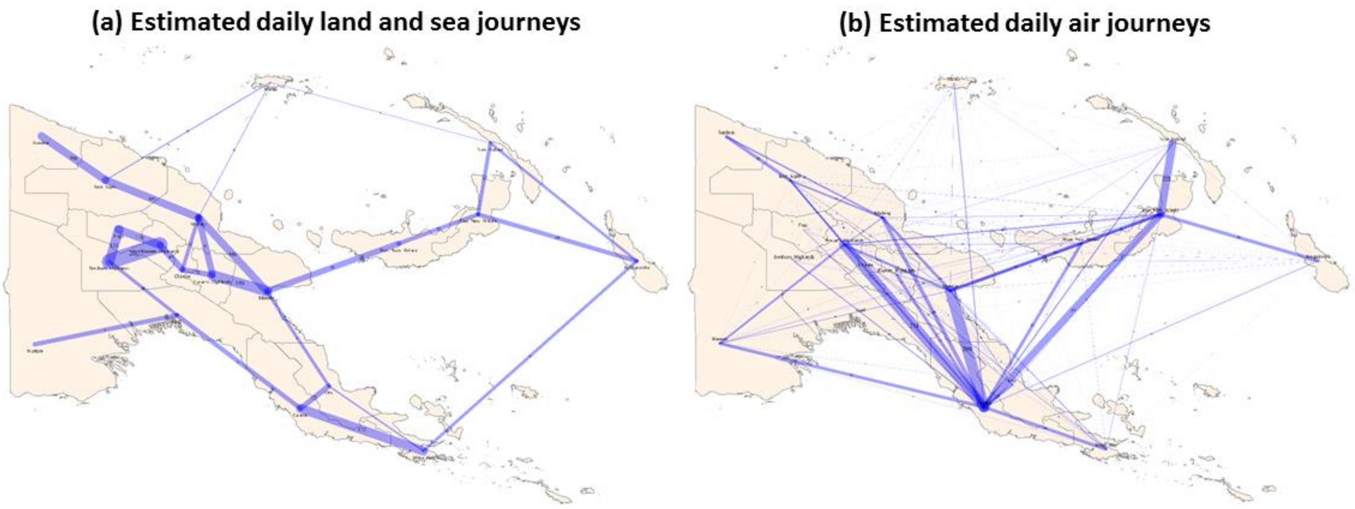

Supplementary Figure 7: Estimated daily inter-provincial journeys by **(a)** land and sea, and **(b)** air.

It is assumed that when an individual travels from their home province to another province, they stay for an average of 14 days. In particular it is assumed that the duration of journeys is exponentially distributed so that individuals return home at a constant rate.

A limitation to modelling inter-provincial travel in this manner is that we do not account for individuals who live close to the borders between two provinces and who may regularly travel back and forth between provinces.

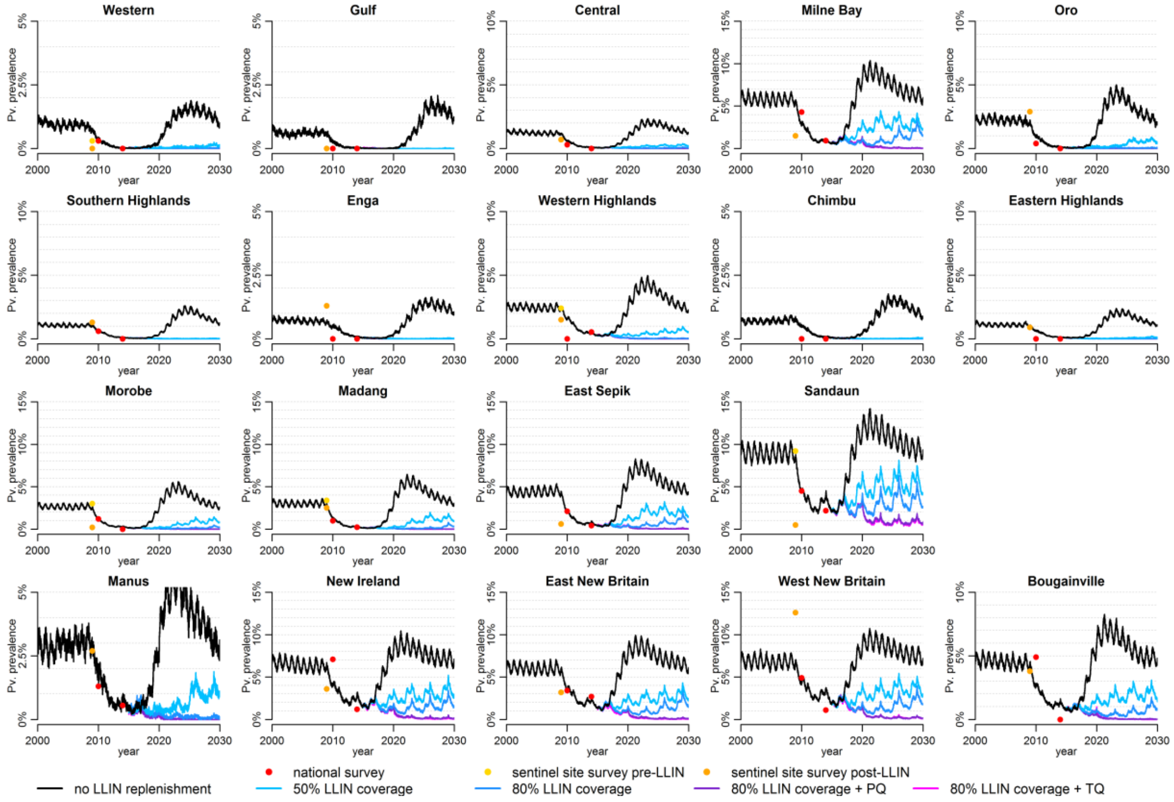

Supplementary Figure 8: Predicted *Pv*PR_{LM} in Papua New Guinean provinces using individual-based model with inter-provincial travel. Data are from household prevalence surveys in randomly selected villages, and surveys from a number of sentinel villages either before or after LLIN distribution. The black curves denote the model predicted scenario if LLINs are not replaced. In the LLIN campaigns, nets are assumed to be distributed every 3 years, with 50% of nets still in use after 19.5 months. Primaquine (PQ) or tafenoquine (TQ) with accompanying G6PD screening are assumed to be include in first line treatment regimens from 2020, with 50% of individuals experiencing a clinical episode of *P. vivax* being tested and treated.

5. Supplementary model simulations

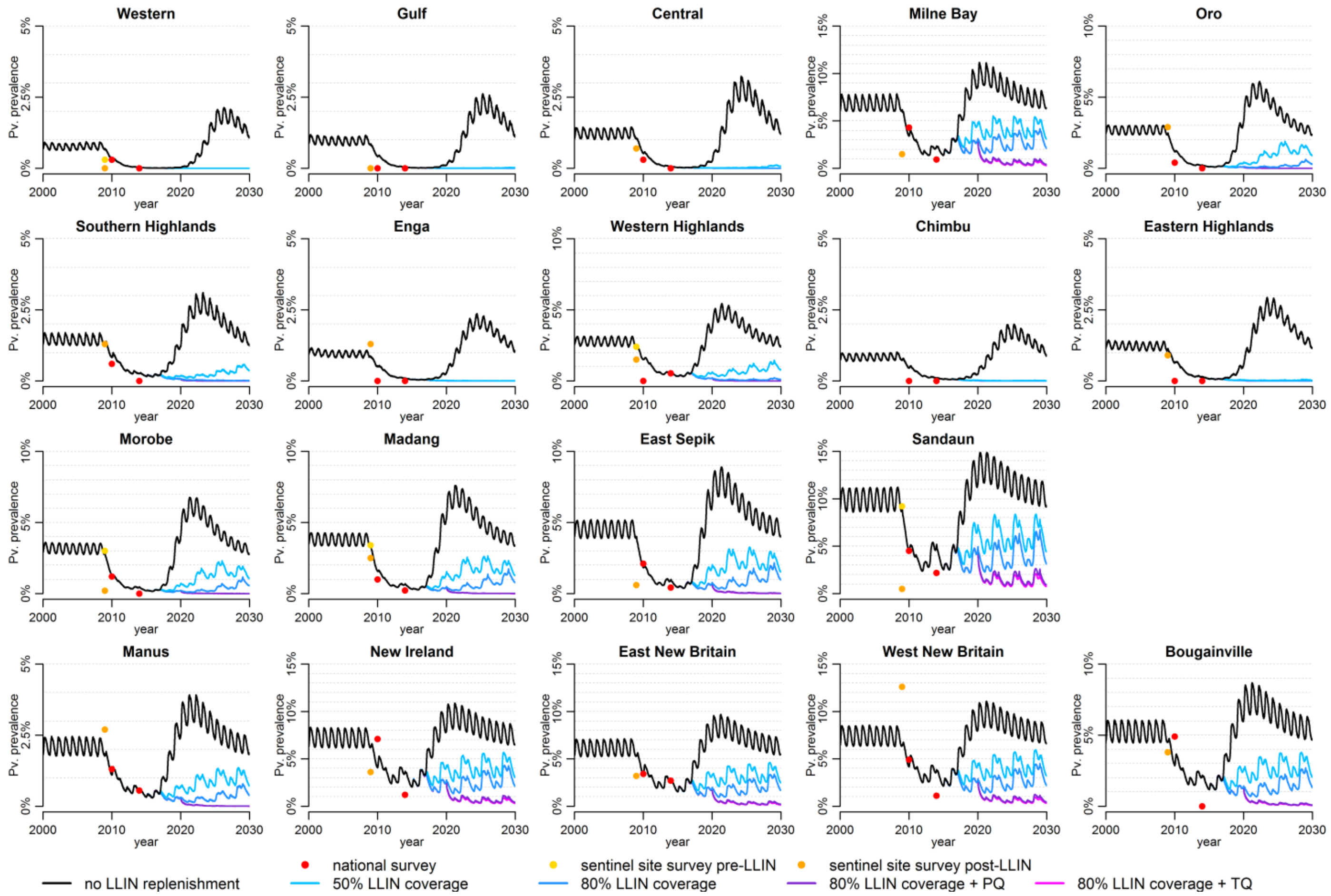

Supplementary Figure 9: Individual-based model predictions of $Pv\text{PR}_{LM}$ in Papua New Guinean provinces under a scenario where LLINs are replaced every 3 years at 50% coverage. In this sensitivity analysis, a reduced rate of loss of adherence is assumed such that 50% of LLINs are lost after 23 months. 50% of symptomatic clinical *P. vivax* cases are assumed to receive treatment with an ACT.

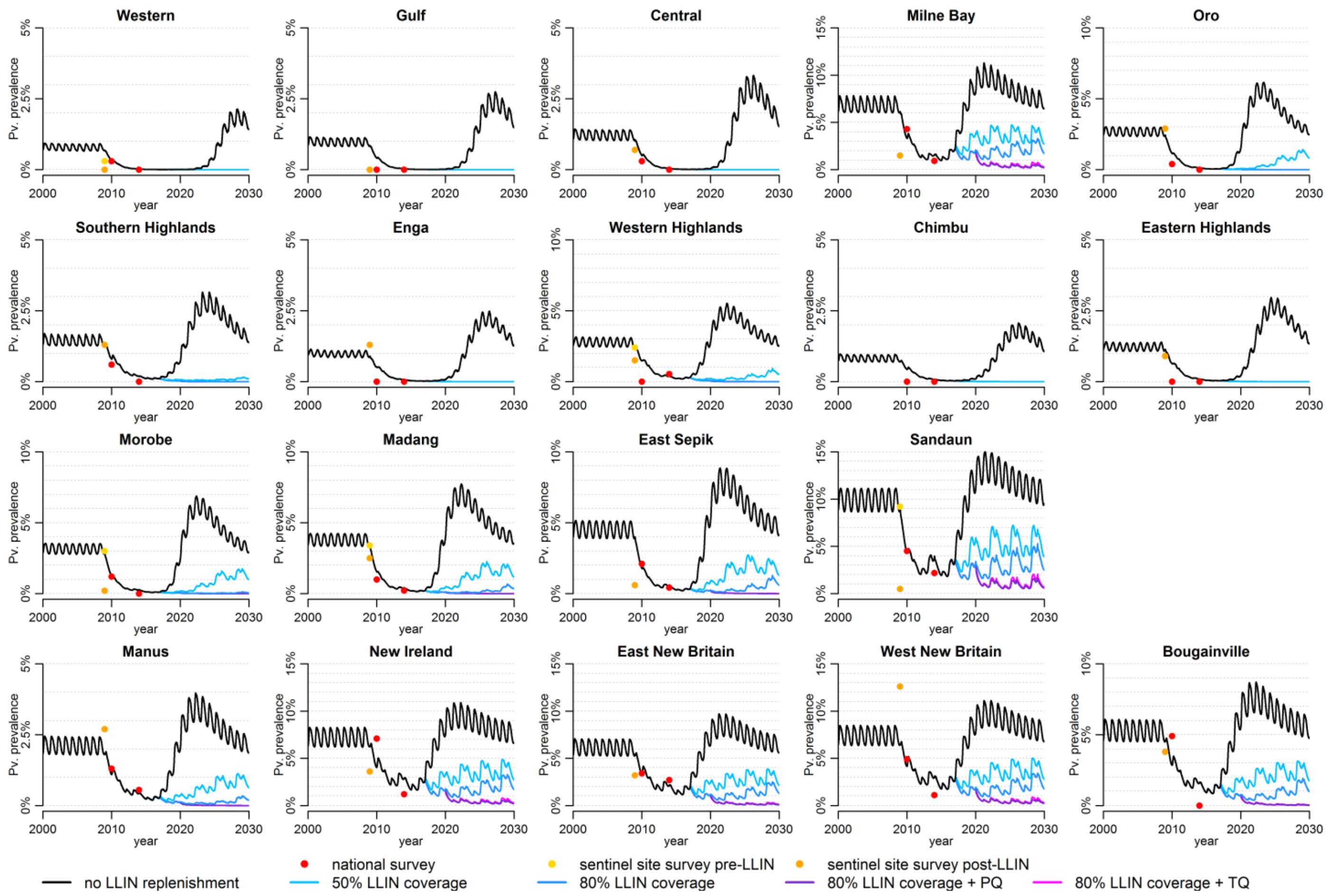

Supplementary Figure 10: Individual-based model predictions of *Pv*PR$_{LM}$ in Papua New Guinean provinces under a scenario where LLINs are replaced every 3 years at 50% coverage. In this sensitivity analysis, a reduced rate of loss of adherence is assumed such that 50% of LLINs are lost after 36 months. 50% of symptomatic clinical *P. vivax* cases are assumed to receive treatment with an ACT.

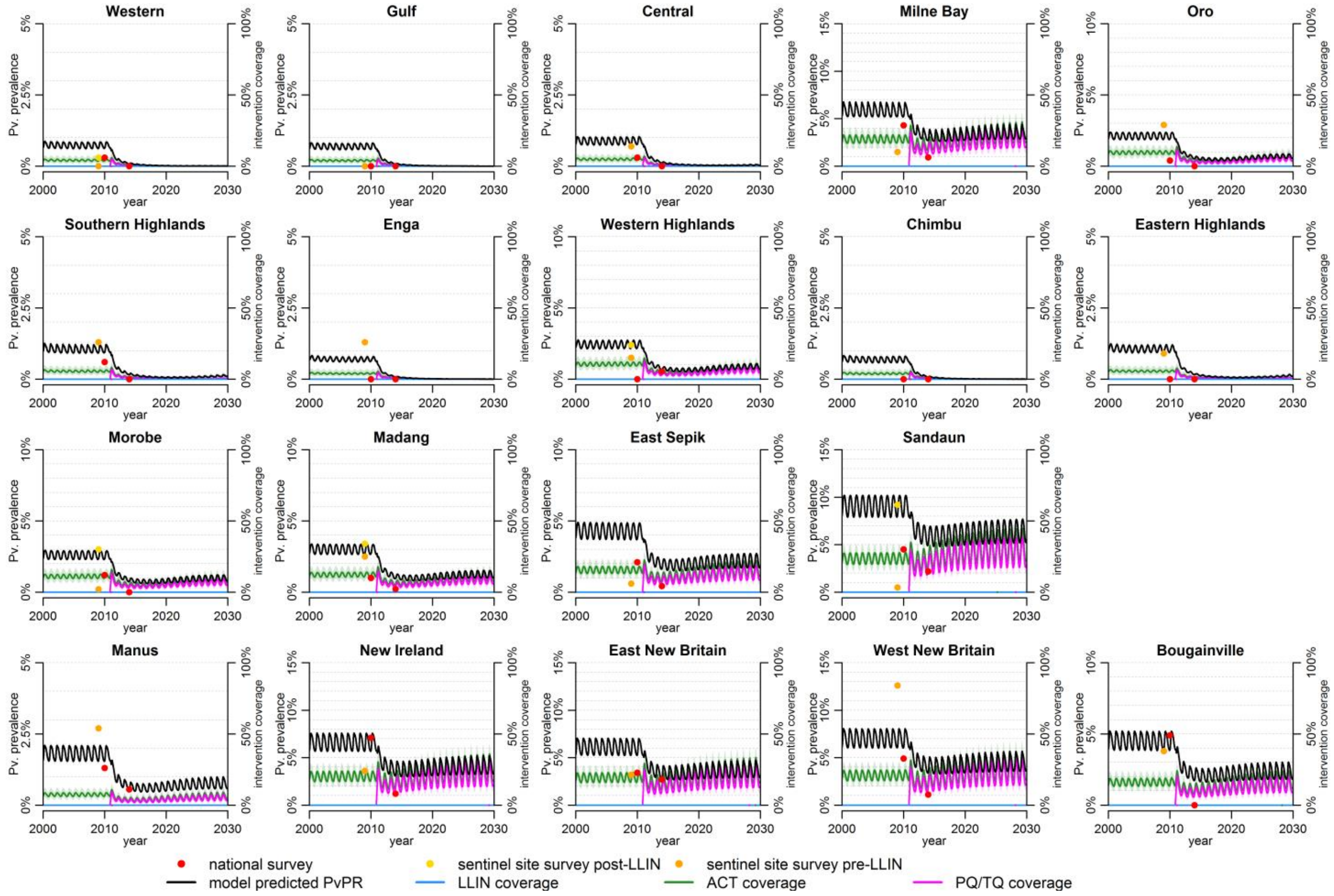

Supplementary Figure 11: Counterfactual simulation where it was assumed that LLINs were not effective and not distributed, and reductions in *Pv*PR_{LM} are instead attributable to an introduction of routine primaquine treatment with screening for G6PD deficiency from 2011.

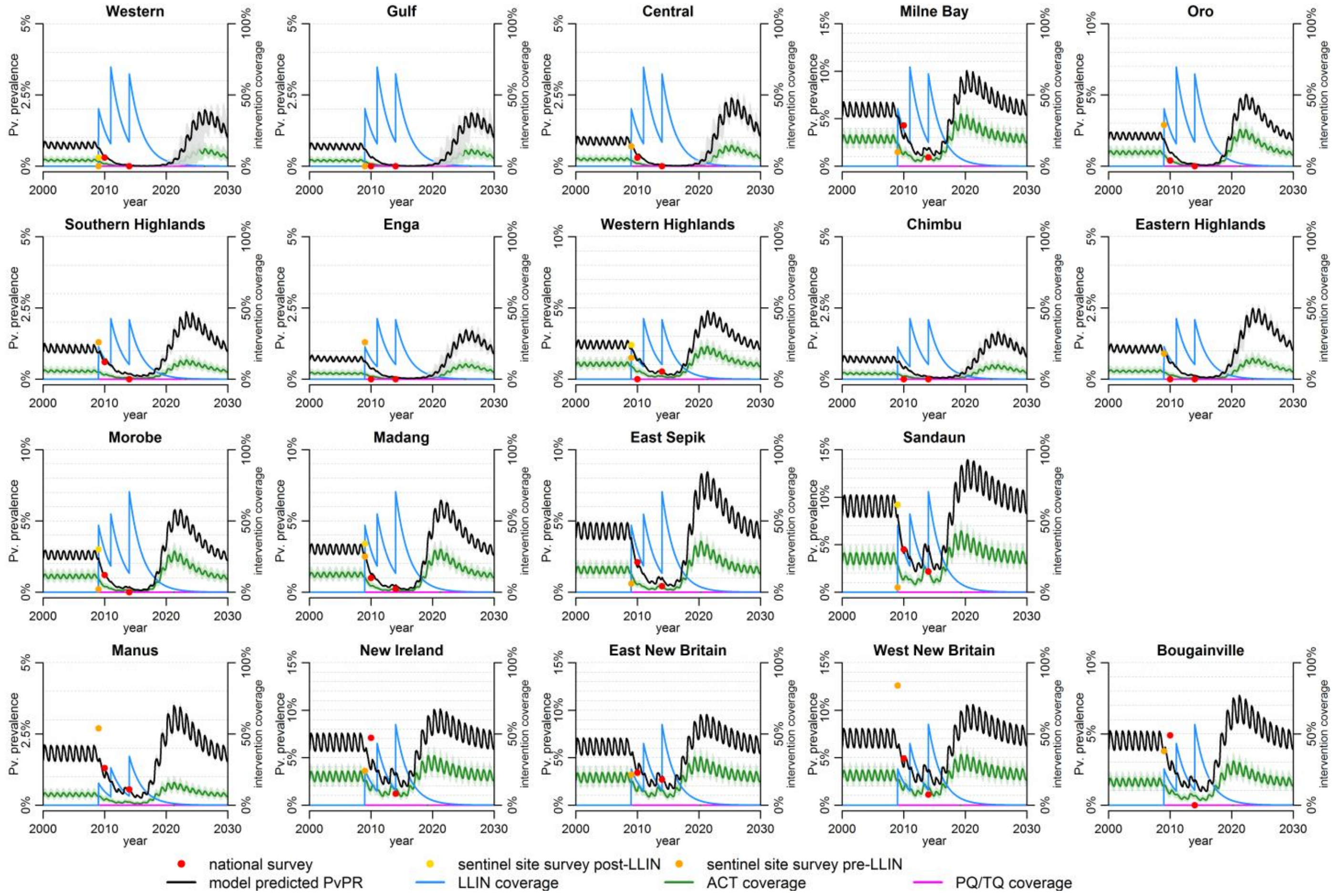

Supplementary Figure 12: Individual-based model predictions of *Pv*PR_{LM} in Papua New Guinean provinces under a scenario where LLINs are not replaced. 50% of symptomatic clinical *P. vivax* cases are assumed to receive treatment with an ACT. The green curve for ACT coverage shows the proportion of individuals receiving treatment in a year. The blue curve for LLIN coverage shows the proportion of individuals sleeping under a bed net.

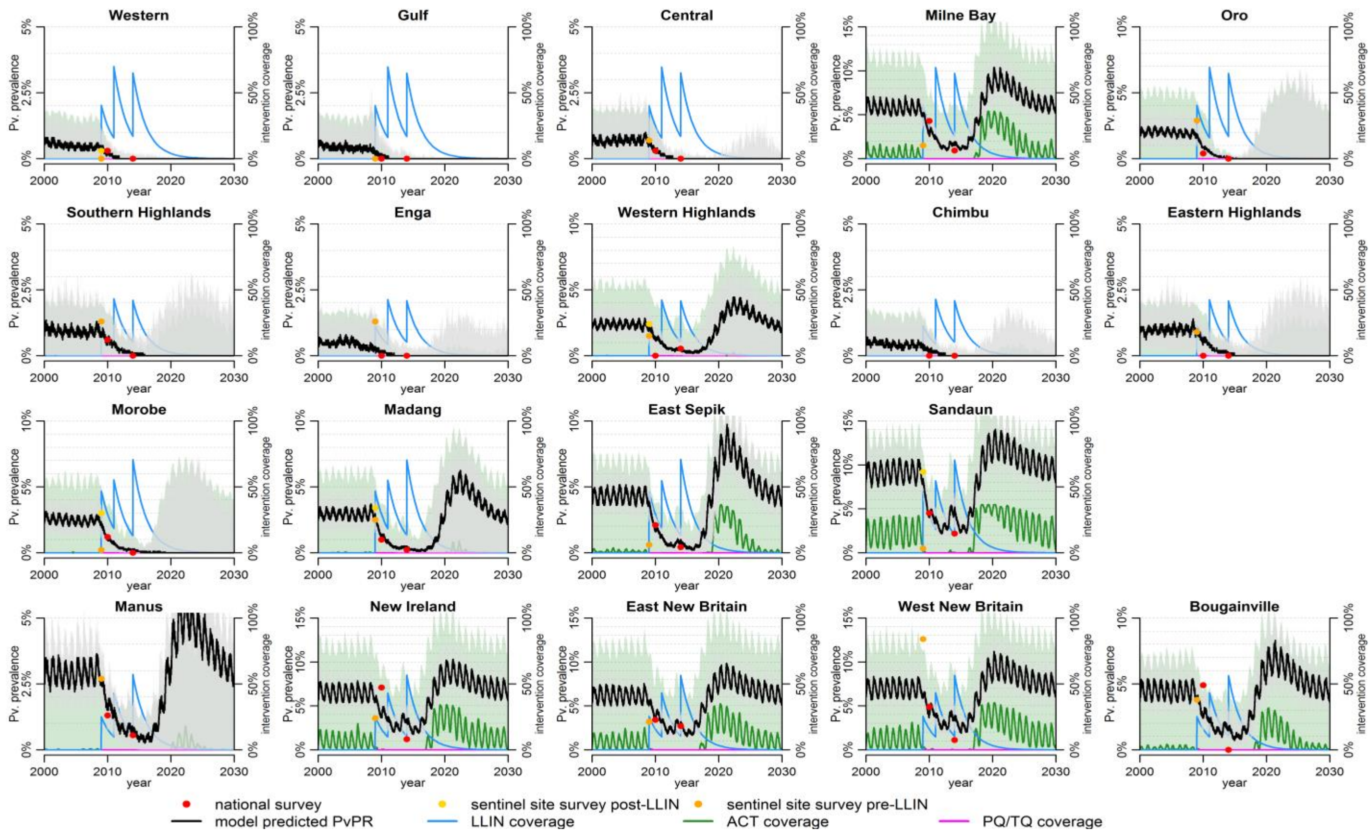

Supplementary Figure 13: Individual-based model predictions of *Pv*PR$_{LM}$ in Papua New Guinean provinces under a scenario where LLINs are not replaced. The simulated population size has been reduced from 100,000 to 1,000 resulting in substantially greater stochasticity. 50% of symptomatic clinical *P. vivax* cases are assumed to receive treatment with an ACT. The green curve for ACT coverage shows the proportion of individuals receiving treatment in a year. The blue curve for LLIN coverage shows the proportion of individuals sleeping under a bed net.

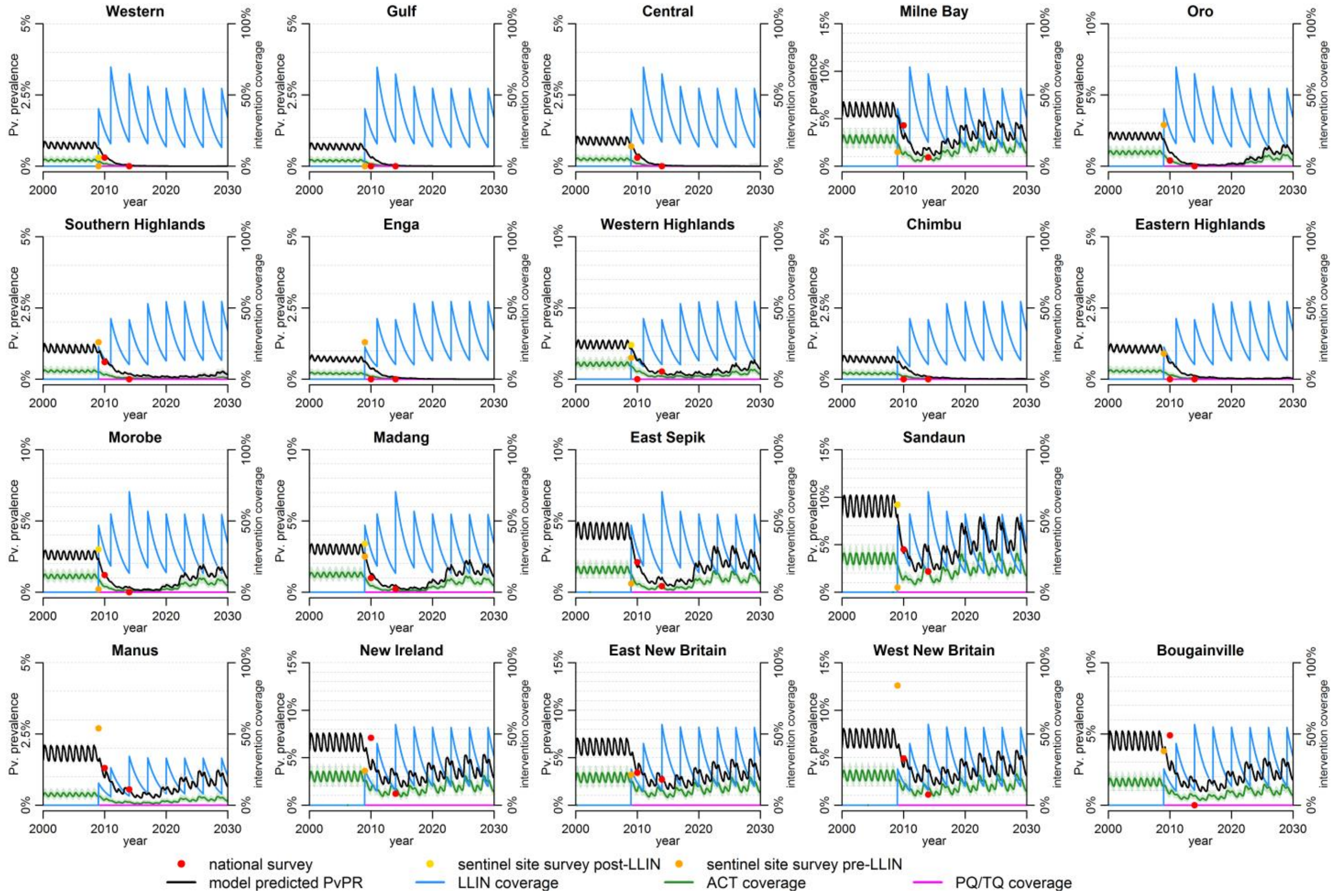

Supplementary Figure 14: Individual-based model predictions of *Pv*PR$_{LM}$ in Papua New Guinean provinces under a scenario where LLINs are replaced every 3 years at 50% coverage. 50% of symptomatic clinical *P. vivax* cases are assumed to receive treatment with an ACT. The green curve for ACT coverage shows the proportion of individuals receiving treatment in a year. The blue curve for LLIN coverage shows the proportion of individuals sleeping under a bed net.

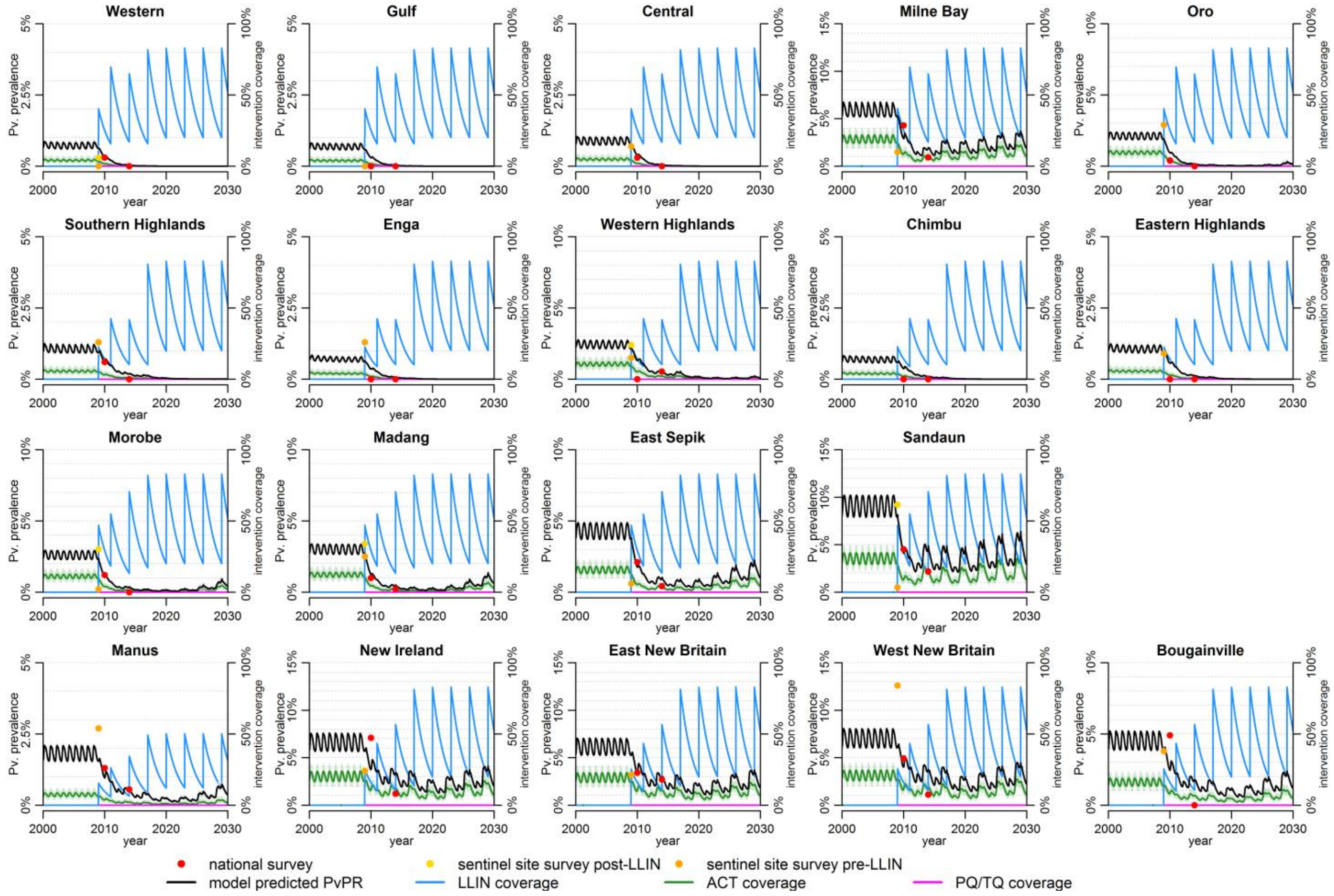

Supplementary Figure 15: Individual-based model predictions of *Pv*PR$_{LM}$ in Papua New Guinean provinces under a scenario where LLINs are replaced every 3 years at 80% coverage. 50% of symptomatic clinical *P. vivax* cases are assumed to receive treatment with an ACT. The green curve for ACT coverage shows the proportion of individuals receiving treatment in a year. The blue curve for LLIN coverage shows the proportion of individuals sleeping under a bed net.

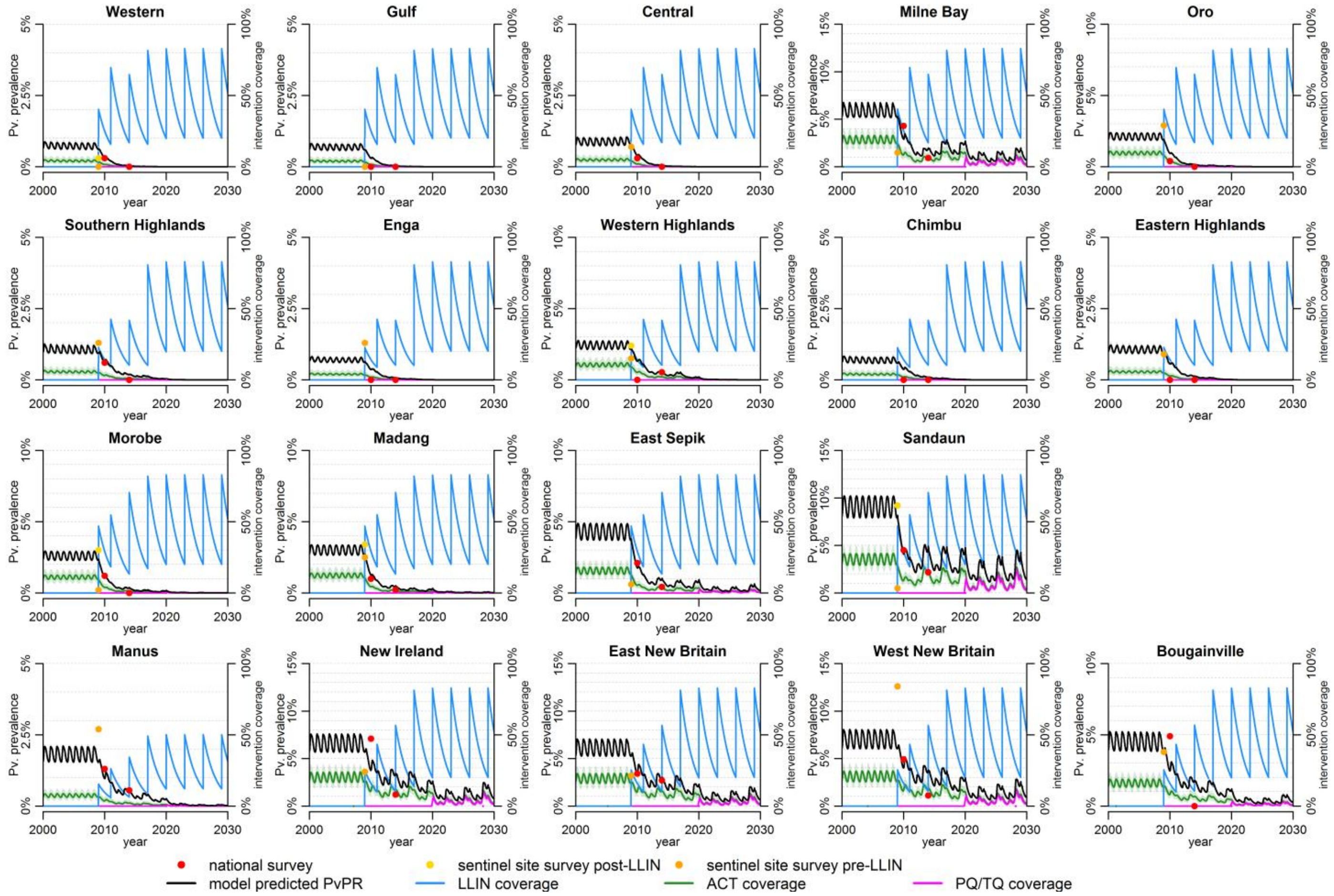

Supplementary Figure 16: Individual-based model predictions of $Pv\text{PR}_{LM}$ in Papua New Guinean provinces under a scenario where LLINs are replaced every 3 years at 80% coverage. 30% of symptomatic clinical *P. vivax* cases are assumed to receive treatment with an ACT and tafenoquine with G6PD screening (introduced from 2020) according to the treatment pathway in Supplementary Figure 5. The green curve for ACT coverage and the pink curve for PQ/TQ coverage shows the proportion of individuals receiving treatment in a year. The blue curve for LLIN coverage shows the proportion of individuals sleeping under a bed net.

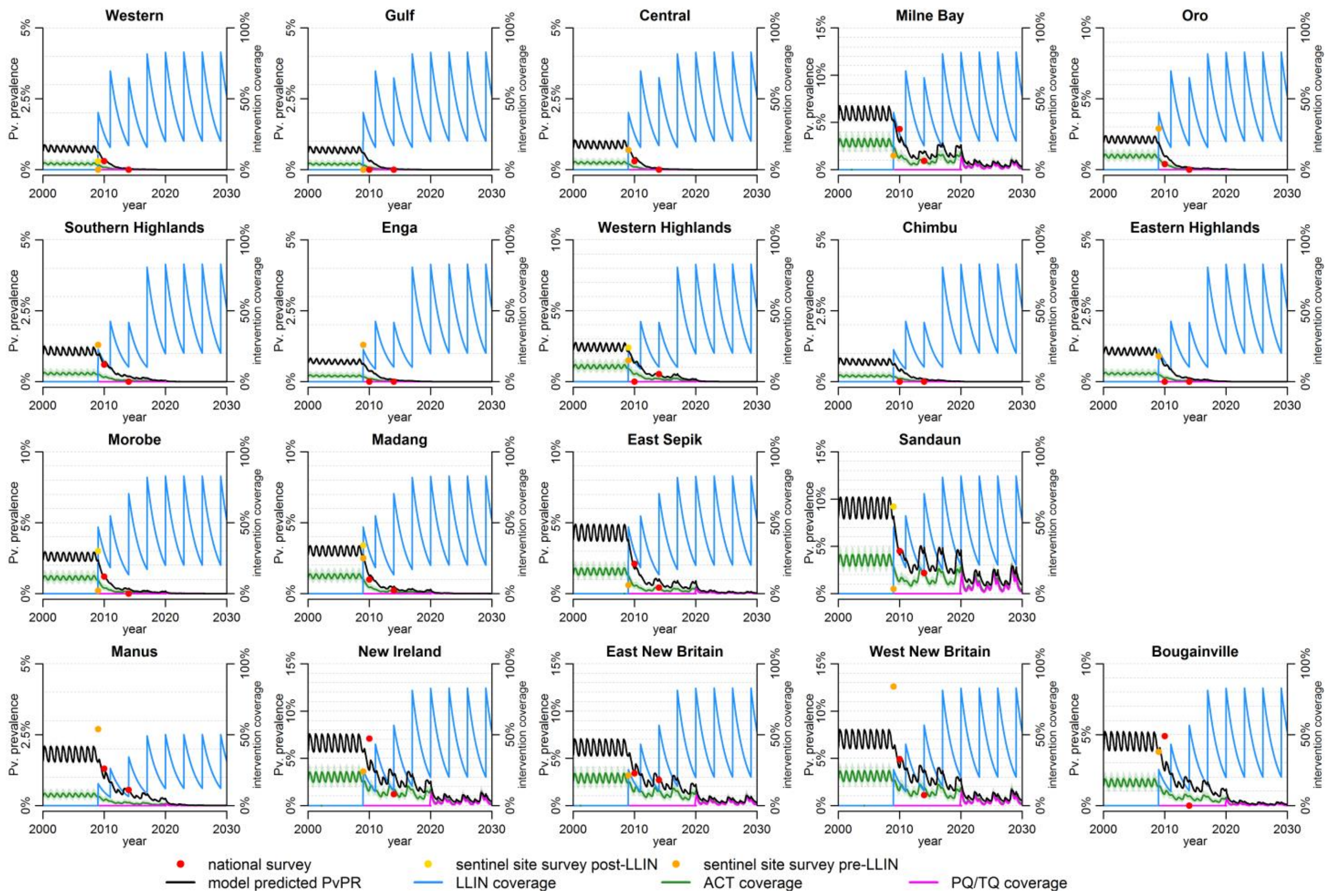

Supplementary Figure 17: Individual-based model predictions of *Pv*PR_{LM} in Papua New Guinean provinces under a scenario where LLINs are replaced every 3 years at 80% coverage. 50% of symptomatic clinical *P. vivax* cases are assumed to receive treatment with an ACT and tafenoquine with G6PD screening (introduced from 2020) according to the treatment pathway in Supplementary Figure 5. The green curve for ACT coverage and the pink curve for PQ/TQ coverage shows the proportion of individuals receiving treatment in a year. The blue curve for LLIN coverage shows the proportion of individuals sleeping under a bed net.

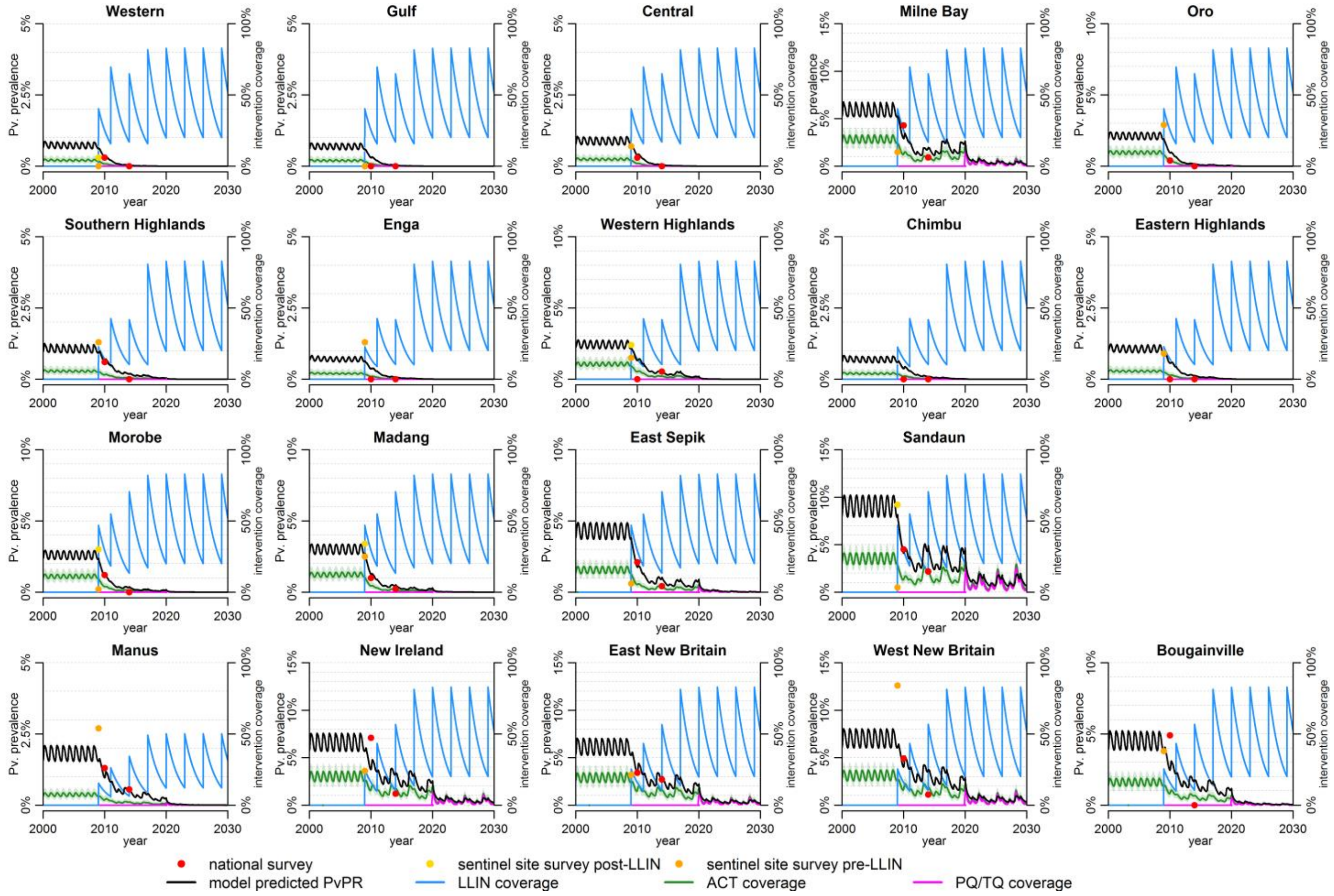

Supplementary Figure 18: Individual-based model predictions of *Pv*PR_{LM} in Papua New Guinean provinces under a scenario where LLINs are replaced every 3 years at 80% coverage. 70% of symptomatic clinical *P. vivax* cases are assumed to receive treatment with an ACT and tafenoquine with G6PD screening (introduced from 2020) according to the treatment pathway in Supplementary Figure 5. The green curve for ACT coverage and the pink curve for PQ/TQ coverage shows the proportion of individuals receiving treatment in a year. The blue curve for LLIN coverage shows the proportion of individuals sleeping under a bed net.

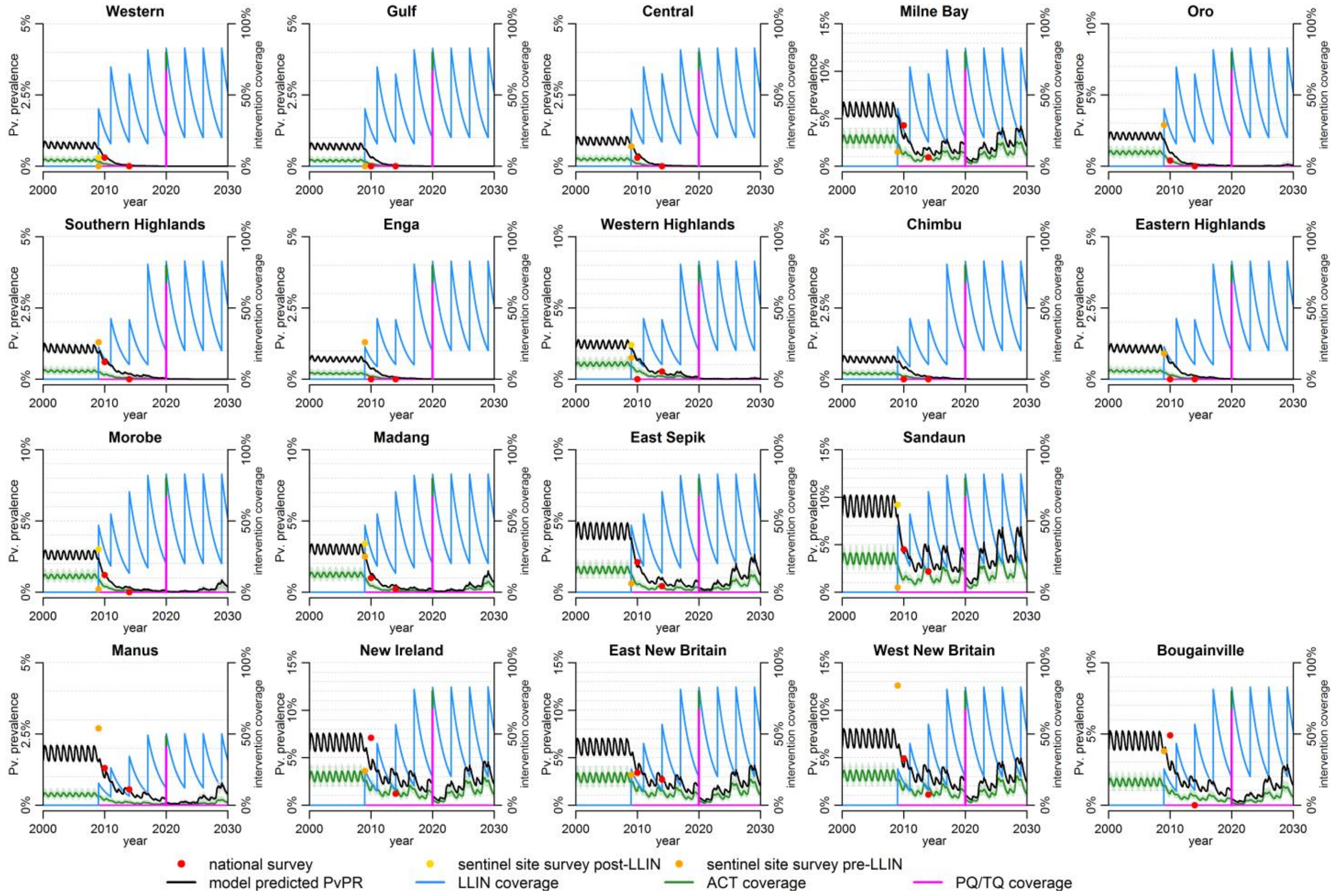

Supplementary Figure 19: Individual-based model predictions of $Pv\mathrm{PR}_{\mathrm{LM}}$ in Papua New Guinean provinces under a scenario where LLINs are replaced every 3 years at 80% coverage. 50% of symptomatic clinical *P. vivax* cases are assumed to receive treatment with an ACT. The green curve for ACT coverage shows the proportion of individuals receiving treatment in a year. The blue curve for LLIN coverage shows the proportion of individuals sleeping under a bed net. A round of mass drug administration (MDA) with tafenoquine and G6PD screening was implemented in 2020 at 80% coverage.

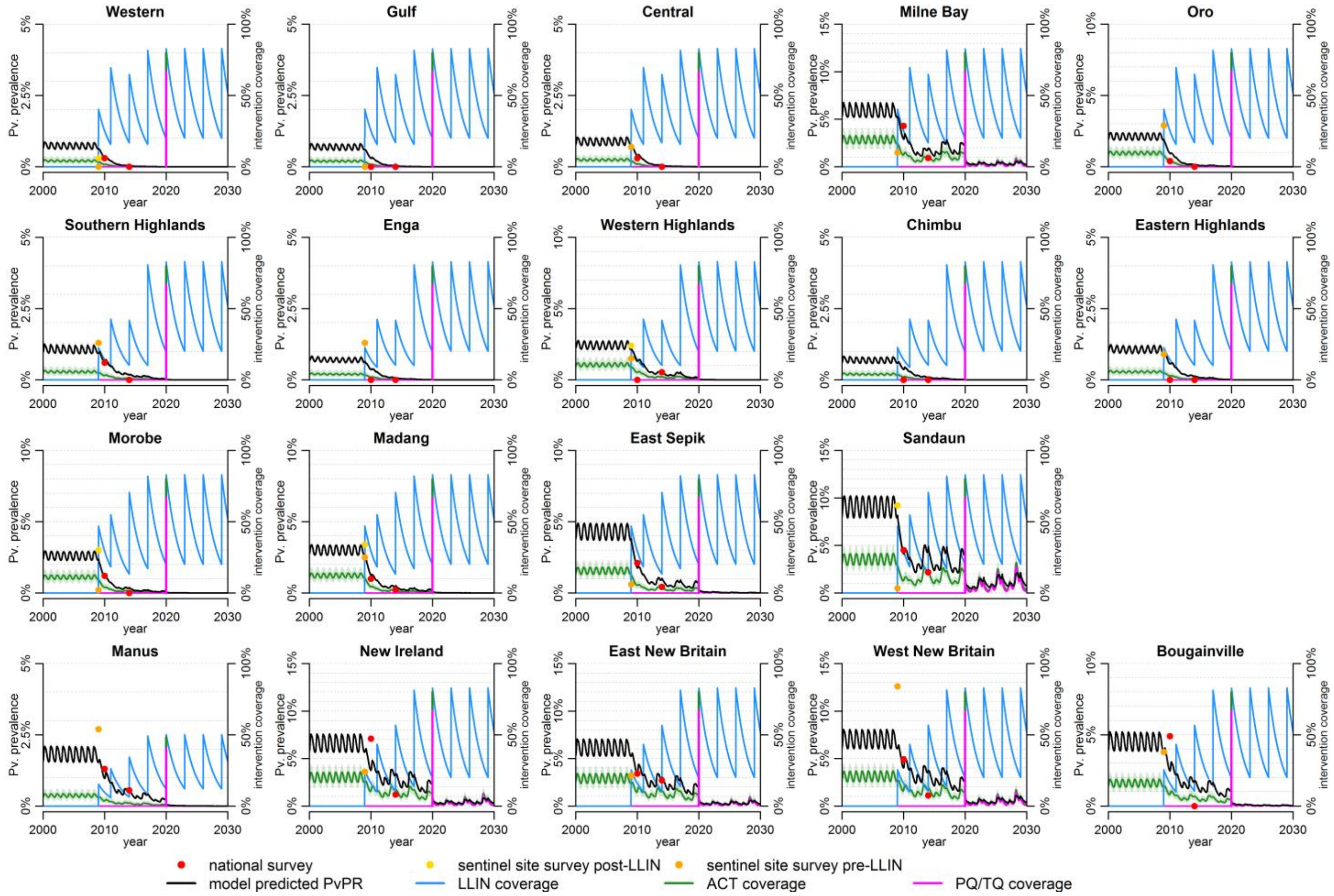

Supplementary Figure 20: Individual-based model predictions of $Pv\text{PR}_{\text{LM}}$ in Papua New Guinean provinces under a scenario where LLINs are replaced every 3 years at 80% coverage. 70% of symptomatic clinical *P. vivax* cases are assumed to receive treatment with an ACT and tafenoquine with G6PD screening (introduced from 2020) according to the treatment pathway in Supplementary Figure 5. The green curve for ACT coverage and the pink curve for PQ/TQ coverage shows the proportion of individuals receiving treatment in a year. The blue curve for LLIN coverage shows the proportion of individuals sleeping under a bed net. A round of mass drug administration (MDA) with tafenoquine and G6PD screening was implemented in 2020 at 80% coverage.

Supplementary References

1. Macdonald G. The analysis of equilibrium in malaria. *Trop. Dis. Bull.* **49**(9): 813–1129 (1952)
2. Smith DL, Battle KE, Hay SI, Barker CM, Scott TW, McKenzie FE. Ross, Macdonald, and a Theory for the Dynamics and Control of Mosquito-Transmitted Pathogens. *PLoS Pathog.* **8**(4): e1002588 (2012)
3. Reiner RC,et al. A systematic review of mathematical models of mosquito-borne pathogen transmission: 1970–2010. *J. Roy. Soc. Interface* **10**:81 (2013)
4. Mandal S, Sarkar RR, Sinha S. Mathematical models of malaria - a review. *Malaria J.* **10**:202 (2011)
5. White MT, Shirreff G, Karl S, Ghani AC, Mueller I. Variation in relapse frequency and the transmission potential of *Plasmodium vivax* malaria. *Proc. Roy. Soc. B* **283**:1827 (2016)
6. White MT, Karl S, Battle KE, Hay SI, Mueller I, Ghani AC. Modelling the contribution of the hypnozoite reservoir to *Plasmodium vivax* transmission. *eLife.* 3:10.7554 (2014)
7. Griffin JT, et al. Reducing *Plasmodium falciparum* Malaria Transmission in Africa: A Model-Based Evaluation of Intervention Strategies. *PLoS Med.* **7**(8):e1000324 (2010)
8. Carnevale P, Frezil JL, Bosseno MF, Le Pont F, Lancien J. The aggressiveness of *Anopheles gambiae* A in relation to the age and sex of the human subjects [*In French*]. *Bull. World. Health Organ.* **56**(1): 147-54. (1978)
9. Port GR, Boreham PFL, Bryan JH. The Relationship of Host Size to Feeding by Mosquitos of the *Anopheles-Gambiae Giles* Complex (Diptera, Culicidae). *Bull. Entomol. Res.* **70**(1): 133-144. (1980)
10. Keating J, et al. *Anopheles gambiae* s.l. and *Anopheles funestus* mosquito distributions at 30 villages along the Kenyan coast. *J. Med. Entomol.* **42**(3): 241-246 (2005)
11. Smith T, Charlwood JD, Takken W, Tanner M, Spiegelhalter DJ. Mapping the densities of malaria vectors within a single village. *Acta Trop.* **59**(1): 1-18. (1995)
12. Cooper RD, Waterson DGE, Frances SP, Beebe NW, Sweeney AW. Speciation and distribution of the members of the *Anopheles punctulatus* group (Diptera: Culicidae) in Papua New Guinea. *J. Med. Entomol.* **39**(1): 16–27 (2002)
13. White MT, Griffin JT, Churcher TS, Ferguson NM, Basáñez MG, Ghani AC. Modelling the impact of vector control interventions on *Anopheles gambiae* population dynamics. *Parasit. Vectors* **14**:153 (2011)
14. Battle KE, et al. Defining the relationship between *Plasmodium vivax* parasite rate and clinical disease. *Malar. J.* **14**:191 (2015)
15. Robinson LJ, et al. Strategies for Understanding and Reducing the *Plasmodium vivax* and *Plasmodium ovale* Hypnozoite Reservoir in Papua New Guinean Children: A Randomised Placebo-Controlled Trial and Mathematical Model. *PLOS Med.* **12**(10):e1001891 (2015)
16. Brooks SP, Gelman A. General Methods for Monitoring Convergence of Iterative Simulations. *J. Comp. Graph. Stats.* **7**: 434–455. (1997)
17. Keeling M, Rohani P. Modeling infectious diseases in humans and animals. *Princeton Univ. Press., Princeton.* (2008)
18. Le Menach A, et al. An elaborated feeding cycle model for reductions in vectorial capacity of night-biting mosquitoes by insecticide-treated nets. *Malar. J.* **6**:10 (2006)
19. Kilian A, et al. Universal coverage with insecticide-treated nets – applying the revised indicators for ownership and use to the Nigeria 2010 malaria indicator survey data. *Malar. J.* **12**:314 (2013)
20. Katusele M, Gideon G, Thomsen EK, Siba PM, Hetzel MW, Reimer LJ. Long-lasting insecticidal nets remain efficacious after five years of use in Papua New Guinea. *P.N.G. Med. J.* **57**(1-4): 86-93 (2014)
21. Okumu FO, et al. A Modified Experimental Hut Design for Studying Responses of Disease-Transmitting Mosquitoes to Indoor Interventions: The Ifakara Experimental Huts. *PLoS ONE* **7**(2): e30967 (2012)
22. Battle KE, et al. Treatment-seeking rates in malaria endemic countries. *Malar. J.* **15**:20 (2016)
23. Hetzel MW, et al. Prevalence of malaria across Papua New Guinea after initial roll-out of insecticide-treated mosquito nets. *Trop. Med. Int. Health.* **20**(12): 1745-1755 (2015)
24. Hetzel MW, et al. Insecticide-treated nets and malaria prevalence, Papua New Guinea, 2008-2014. *Bull. World. Health Organ.* **95**: 695-705 (2017)
25. Bugoro H, et al. Bionomics of the malaria vector Anopheles farauti in Temotu Province, Solomon Islands: issues for malaria elimination. *Malar. J.* **10**:133 (2011a)
26. Reimer LJ, et al. Malaria transmission dynamics surrounding the first nationwide long-lasting insecticidal net distribution in Papua New Guinea. *Malar. J.* **15**:25 (2016)
27. Waltmann A, et al. High Rates of Asymptomatic, Submicroscopic *Plasmodium vivax* Infection and Disappearing *Plasmodium falciparum* Malaria in an Area of Low Transmission in Solomon Islands. *PLoS Negl. Trop. Dis.* **9**(5): e0003758 (2015)

28. Senn N, et al. Population Hemoglobin Mean and Anemia Prevalence in Papua New Guinea: New Metrics for Defining Malaria Endemicity? *PLoS One* **5**(2): e9375 (2010)
29. Mueller I, et al. Three different *Plasmodium* species show similar patterns of clinical tolerance of malaria infection. *Malar. J.* **8**:158 (2009)
30. Genton B, et al. The epidemiology of malaria in the Wosera area, East Sepik Province, Papua New Guinea, in preparation for vaccine trials. I. Malariometric indices and immunity. *Ann. Trop. Med. Parasitol.* **89**(4): 359-76. (1995)
31. Kasehagen LJ, et al. Changing patterns of *Plasmodium* blood-stage infections in the Wosera region of Papua New Guinea monitored by light microscopy and high throughput PCR diagnosis. *Am. J. Trop. Med. Hyg.* **75**(4): 588–596 (2006)
32. Koepfli C, et al. Blood-Stage Parasitaemia and Age Determine *Plasmodium falciparum* and *P. vivax* Gametocytaemia in Papua New Guinea. *PLoS ONE* **10**(5): e0126747. (2015)
33. Lin E, et al. Differential Patterns of Infection and Disease with *P. falciparum* and *P. vivax* in Young Papua New Guinean Children. *PLoS ONE* **5**(2): e9047 (2010)
34. Michon P, et al. The risk of malarial infections and disease in Papua New Guinean children. *Am. J. Trop. Med. Hyg.* **76**(6): 997–1008 (2007)
35. Kasehagen LJ, et al. Reduced *Plasmodium vivax* Erythrocyte Infection in PNG Duffy-Negative Heterozygotes. *PLoS ONE* **2**(3): e336 (2007)
36. Herrera S, et al. Consistent Safety and Infectivity in Sporozoite Challenge Model of *Plasmodium vivax* in Malaria-Naive Human Volunteers. *Am. J. Trop. Med. Hyg.* **84**(Suppl 2): 4–11 (2011)
37. Pukrittayakamee S, Imwong M, Singhasivanon P, Stepniewska K, Day NJ, White NJ. Effects of different antimalarial drugs on gametocyte carriage in *P. vivax* malaria. *Am. J. Trop. Med. Hyg.* **79**(3): 378-84 (2008).
38. Kiattibutr K, et al. Infectivity of symptomatic and asymptomatic *Plasmodium vivax* infections to a Southeast Asian vector, *Anopheles dirus*. *Int. J. Parasitol.* **47**(2-3): 163-170 (2017)
39. Clements AN, Paterson GD. The analysis of mortality and survival rates in wild populations of mosquitoes. *J. Applied Ecol.* **18**(2): 373-399 (1981)
40. Gething PW, et al. Modelling the global constraints of temperature on transmission of *Plasmodium falciparum* and *P. vivax*. *Parasit. Vectors* **4**:92 (2011)
41. Charlwood JD, Dagoro H, Paru R. Blood-feeding and resting behaviour in the *Anopheles punctulatus* Donitz complex (Diptera: Culicidae) in Papua New Guinea. *Bull. Entomol. Res.* **75**(3): 463-475 (1985)
42. Charlwood JD, Graves PM, Alpers MP. The ecology of the *Anopheles punctulatus* group of mosquitoes from Papua New Guinea: a review of recent work. *P.N.G. Med. J.* **29**(1):19-26 (1986)
43. Killeen GF, Smith TA. Exploring the contributions of bed nets, cattle, insecticides and excitorepellency to malaria control: a deterministic model of mosquito host-seeking behaviour and mortality. *Trans. Roy. Soc. Trop. Med. Hyg.* **101**(9-4): 867-880 (2007)
44. Keven JB, et al. Plasticity of host selection by malaria vectors of Papua New Guinea. *Parasit. Vectors* **10**:95 (2017)
45. Russell TL, et al. Determinants of host feeding success by *Anopheles farauti*. *Malar. J.* **15**:152 (2016a)
46. Killeen GF, McKenzie FE, Foy BD, Schieffelin C, Billingsley PF, Beier JC. A simplified model for predicting malaria entomologic inoculation rates based on entomologic and parasitologic parameters relevant to control. *Am. J. Trop. Med. Hyg.* **62**(5): 535-544 (2000)
47. Hii JL, et al. Spatial and temporal variation in abundance of *Anopheles* (Diptera:Culicidae) in a malaria endemic area in Papua New Guinea. *J. Med. Entomol.* **34**(2): 193-205 (1997)
48. Burkot TR, Graves PM. The value of vector-based estimates of malaria transmission. *Ann. Trop. Med. Parasitol.* **89**(2): 125-134 (1995)
49. Burkot TR, et al. Effects of untreated bed nets on the transmission of *Plasmodium falciparum*, *P. vivax* and *Wuchereria bancrofti* in Papua New Guinea. *Trans. Roy. Soc. Trop. Med. Hyg.* **84**(6): 773-779 (1990a)
50. Burkot TR, Graves PM, Paru R, Battistutta D, Barnes A, Saul A. Variations in malaria transmission rates are not related to *anopheline* survivorship per feeding cycle. *Am. J. Trop. Med. Hyg.* **43**(4): 321-7 (1990b)
51. Burkot TR, Dye C, Graves PM. An analysis of some factors determining the sporozoite rates, human blood indexes, and biting rates of members of the *Anopheles punctulatus* complex in Papua New Guinea. *Am. J. Trop. Med. Hyg.* **43**(4): 321-327 (1989)
52. Charlwood JD, Graves PM. The effect of permethrin-impregnated bednets on a population of *Anopheles farauti* in coastal Papua New Guinea. *Med. Vet. Entomol.* **1**(3): 319-327 (1987)

53. Russell TL, et al. Frequent blood feeding enables insecticide-treated nets to reduce transmission by mosquitoes that bite predominately outdoors. *Malar. J.* **15**:156 (2016b)
54. Russell TL, et al. *Anopheles farauti* is a homogeneous population that blood feeds early and outdoors in the Solomon Islands. *Malar. J.* **15**:151 (2016c)
55. Bugoro H, et al. The bionomics of the malaria vector *Anopheles farauti* in Northern Guadalcanal, Solomon Islands: issues for successful vector control. *Malar. J.* **13**:56 (2014)
56. Bugoro H, et al. Changes in vector species composition and current vector biology and behaviour will favour malaria elimination in Santa Isabel Province, Solomon Islands. *Malar. J.* **10**:287 (2011b)
57. Thomsen EK, et al. Mosquito Behavior Change After Distribution of Bednets Results in Decreased Protection Against Malaria Exposure. *J. Inf. Dis.* **215**(5): 790-797 (2017)
58. Samarawickrema WA, Parkinson AD, Kere N, Galo O. Seasonal abundance and biting behaviour of *Anopheles punctulatus* and *An. koliensis* in Malaita Province, Solomon Islands, and a trial of permethrin impregnated bednets against malaria transmission. *Med. Vet. Entomol.* **6**(4): 371-378 (1992)
59. Howes RE, et al. G6PD Deficiency Prevalence and Estimates of Affected Populations in Malaria Endemic Countries: A Geostatistical Model-Based Map. *PLOS Med.* **9**(11):e1001339 (2012)
60. Karunajeewa HA, et al. A Trial of Combination Antimalarial Therapies in Children from Papua New Guinea. *New Eng. J. Med.* **359**(24): 2545-2557 (2008)
61. John GK, et al. Primaquine radical cure of *Plasmodium vivax*: a critical review of the literature. *Malar. J.* **11**:280 (2012)
62. Llanos-Cuentas A, et al. Tafenoquine plus chloroquine for the treatment and relapse prevention of *Plasmodium vivax* malaria (DETECTIVE): a multicentre, double-blind, randomised, phase 2b dose-selection study. *Lancet*. **383**(9922):1049-58 (2014)
63. Afifi SED, Spencer M, Hudson P, Tavil NW. Biting prevalence and malaria transmission patterns in the *Anopheles punctulatus* complex (Diptera: Culicidae) in Papua New Guinea. *Aust. J. Exp. Biol. Med. Sci.* **58**(1): 1-17 (1980)
64. Kere NK, Parkinson AD, Samarawickrema WA. The effect of permethrin impregnated bednets on the incidence of *Plasmodium falciparum*, in children of North Guadalcanal, Solomon Islands. *S.E. Asian. J. Trop. Med. Pub. Health.* **24**(1): 130-137 (1993)
